# The diverse roles of macrophages in metabolic inflammation and its resolution

**DOI:** 10.3389/fcell.2023.1147434

**Published:** 2023-03-13

**Authors:** Aleepta Guha Ray, Oluwatomilayo Patience Odum, Destini Wiseman, Ada Weinstock

**Affiliations:** Section of Genetic Medicine, Department of Medicine, The University of Chicago, Chicago, IL, United States

**Keywords:** macrophage, obesity, inflammation, atherosclerosis, non-alcoholic fatty liver disease, diabetes, neuroinflammation

## Abstract

Macrophages are one of the most functionally diverse immune cells, indispensable to maintain tissue integrity and metabolic health. Macrophages perform a myriad of functions ranging from promoting inflammation, through inflammation resolution to restoring and maintaining tissue homeostasis. Metabolic diseases encompass a growing list of diseases which develop from a mix of genetics and environmental cues leading to metabolic dysregulation and subsequent inflammation. In this review, we summarize the contributions of macrophages to four metabolic conditions–insulin resistance and adipose tissue inflammation, atherosclerosis, non-alcoholic fatty liver disease and neurodegeneration. The role of macrophages is complex, yet they hold great promise as potential therapies to address these growing health concerns.

## Introduction

**FIGURE 1:**
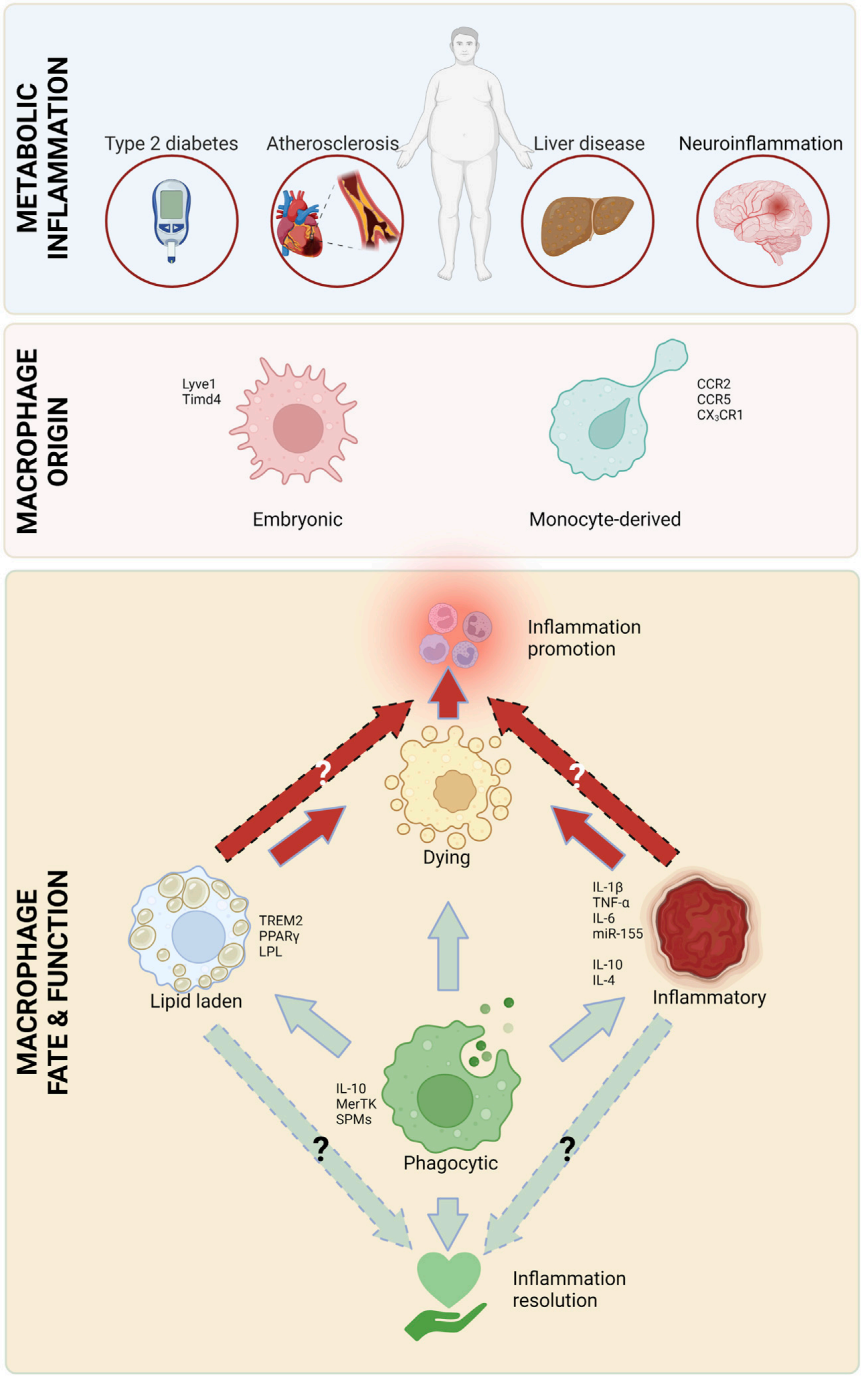
Major macrophage attributes in metabolic inflammation. Macrophages, originating from circulating monocytes and embryonic tissues play a critical role in all types of metabolic inflammation. There are several types of macrophages that are involved in inflammation initiation and heightening (red arrows), as well as resolution (green arrows). Created with Biorender.com.

Obesity is a rapidly growing danger that has reached worldwide epidemic proportions, with half of the US population predicted to be obese by 2030 (Wang et al., 2011). Obesity is an independent risk factor and a comorbid condition to many diseases, including atherosclerotic cardiovascular disease (Rocha and Libby, 2009; Powell-Wiley et al., 2021), diabetes (Barnes, 2011), steatohepatitis (Li et al., 2016) and neurodegenerative diseases (Pugazhenthi et al., 2017). All these diseases are characterized by chronic inflammation involving macrophages as mediators of inflammation. There are multiple overlaps in the pathophysiology of these conditions, including infiltrating monocytes and their differentiation into macrophages, subsequently resulting in uptake of excess lipids, followed by cell retention and death. Inflammation is at the heart of obesity-related conditions and macrophages have a complicated role in both the promotion and alleviation of this inflammation (Figure 1).

Our knowledge about macrophages has evolved from them being immunological first responders to being integral players in tissue homeostasis, and major regulators of systemic and local tissue metabolism. In this way, macrophages play a critical role in balancing the immune response and ensuring that inflammation is resolved in a timely and appropriate manner. Here, we highlight the most important functions of macrophages, including phagocytosis, lipid uptake and excretion, and the production of multitude of inflammation regulating mediators, and how they contribute to the pathogenesis and resolution of metabolic diseases.

### Macrophage origin

Macrophages can be divided into two main types based on their origin: resident and newly recruited (monocyte-derived). Both types of macrophages play important, yet distinct roles in the immune system and the maintenance of tissue health. Resident macrophages are permanently present in tissues. These cells are derived from embryonic tissues (yolk sac and the aorta-gonad-mesonephros-fetal liver axis (Yona et al., 2013; Gomez Perdiguero et al., 2015; Hoeffel et al., 2015; Wu and Hirschi, 2020)) and remain in the tissue throughout the lifetime of the organism. Resident macrophages are important for maintaining the normal tissue function and can also respond rapidly to tissue damage. Tissue macrophages are often maintained by local proliferation, although in some tissues their maintenance requires replenishment from circulating monocytes [reviewed in (Gordon and Pluddemann, 2017)]. Monocyte-derived macrophages, on the other hand, are produced from circulating monocytes that can migrate to tissues when needed. In some situations, monocytes can assume a tissue-resident macrophage phenotype (Zhao et al., 2018). Once in the tissue, these cells differentiate into macrophages and play a key role in the immune response. Monocyte-derived macrophages can be found in almost all tissues in the body and are particularly important for responding to infections and other types of tissue damage. Macrophage numbers in tissues are regulated by dynamic processes, such as recruitment (Swirski et al., 2007; Tacke et al., 2007; Rahman et al., 2017), proliferation (Robbins et al., 2013), retention/stasis (van Gils et al., 2012; Wanschel et al., 2013; Ramkhelawon et al., 2014; Aziz et al., 2017), egress (Bradfield et al., 2016) and apoptosis (Linton et al., 2016) (reviewed in (Weinstock and Fisher, 2019)). In this review we will mainly focus on the differences between embryonic and monocyte-derived macrophages, and will, thus, mostly mention aspects of proliferation/survival and recruitment.

### Lipid handling

Macrophages can take up and break down various types of lipids and lipid particles, including low-density lipoprotein (LDL), high-density lipoprotein (HDL), very low-density lipoprotein (VLDL), which mainly carry triglycerides and cholesterol. Once lipid particles are engulfed, they are broken down, and the resulting products are either used by the macrophage for energy or are further processed and released back into the bloodstream or tissue (Remmerie and Scott, 2018). During inflammation, macrophages typically retain lipids in intracellular lipid droplets, giving these cells the microscopic appearance of containing foam, and were thus termed “foam cells”. Lipid droplets are organelles that store and control the release of lipids. They also regulate inflammation by providing the energy to support several immune processes, such as microbial clearance, production of inflammatory mediators and antigen presentation [reviewed in (den Brok et al., 2018)]. The accumulation of excess fat is a result of multiple factors including dysregulation of influx-efflux pathways and fatty acid oxidation. Importantly, macrophages can clear excess lipids, such as cholesterol, and direct their delivery to the liver for redistribution, breakdown or excretion (Tall and Yvan-Charvet, 2015).

### Cytokines

During the inflammatory response, cytokines are released by immune and non-immune cells and help to recruit other immune cells to the site of injury or infection. Furthermore, they play a key role in regulating the intensity and duration of the inflammatory response. Some of the key cytokines that are involved in promoting metabolic inflammation include interleukin (IL)-1, IL-6, tumor necrosis factor-alpha (TNFα), and interferon (IFN) ɣ (Fujiwara and Kobayashi, 2005). Cytokines such as IL-10 and IL-4 also help to fine-tune the specific immune response needed for inflammation resolution and mediate the repair of tissue damage, to prevent chronic inflammation which can lead to long term tissue damage (Watanabe et al., 2019). Overall, the balance between pro- and anti-inflammatory cytokines is essential for the proper functioning of the immune system.

### Lipid mediators

Omega-3 and omega-6 are a large family of polyunsaturated fatty acids (PUFAs) found in a variety of foods, including fish (mostly omega-3 rich), nuts, seeds, and vegetable oils (mostly omega-6 rich), and are essential for good health. PUFAs have been shown to have anti-inflammatory effects, presumably by inhibiting the production of certain inflammatory molecules, such as thromboxanes and leukotrienes. Furthermore, PUFAs may help to reduce the activity of certain immune cells, such as T and B cells (reviewed in (Calder, 2006)]. Although there is conflicting evidence for the efficacy of either (Ramsden et al., 2013; Ramsden et al., 2016; Abdelhamid et al., 2018), omega-3 is generally considered to have more beneficial effects on health than omega-6 PUFAs, since they are the precursor for many pro-resolving mediators. The most investigated omega-3 fatty acids are eicosapentaenoic acid (EPA) and docosahexaenoic acid (DHA).

REDUCE-IT is a large, randomized controlled trial that evaluated the effectiveness of icosapent ethyl, a form of EPA, in reducing the risk of cardiovascular events in high-risk patients. The results show that treatment with EPA reduces the risk of major cardiovascular events by 25% compared to placebo (Bhatt et al., 2019). This is the first major evidence in humans that lipid mediators may present an effective therapy for metabolic diseases. However, this is an ongoing line of investigation, as a subsequent large clinical trial showed no benefit of a combined formulation of EPA and DHA on major cardiovascular events (Nicholls et al., 2020).

Alongside PUFAs, other lipid mediators have been found to modulate inflammation. Arachidonic acid (which can be derived from PUFAs) is the precursor of several mediators implicated in both inflammation promotion and resolution. In macrophages and other cells, arachidonic acid can be converted to pro-inflammatory leukotrienes (LT), such as LTB_4_, or to pro-resolving mediators, such as lipoxin A_4_ (LXA4) or DHA and resolvins (reviewed in) (Basil and Levy, 2016). Arachidonic acid can also be metabolized by cyclooxygenase (COX; gene name Prostaglandin-endoperoxide synthase, *Ptgs*)-1/2 to prostaglandins (PGs), which were shown to have both pro- and anti-inflammatory properties, according to their location, concentration and cellular target (Vane et al., 1998).

Specialized Pro-resolving Mediators (SPMs) are a class of lipid-derived effectors that are biosynthesized at the site of inflammation and work by activating various G-protein-coupled receptors. They play a critical role in balancing the pro-inflammatory mediators and have been implicated in promoting efferocytosis (Basil and Levy, 2016). SPMs are broadly categorized into lipoxins, resolvins (Rv), protectins and maresins (MaR). They have been found to be protective *in vivo* (in several rodent species)*,* where they promote the clearance of microorganisms, debris and apoptotic cells, and promote tissue repair independently of anti-inflammatory signals (Basil and Levy, 2016).

### MicroRNAs (miR)

miRs are small non-coding RNAs that play a key role in regulating gene expression. They do this by binding to specific target genes and either inhibiting their transcription, translation or influencing their mRNA stability. miRs direct multiple processes by regulating the expression of genes in macrophages which play an important role in maintaining tissue integrity (Liu and Abraham, 2013). Interestingly, miRs are also a mean of cell-cell communication, as they can be transferred through exosomes (Hu et al., 2012) and lipoproteins (Michell and Vickers, 2016).

### Efferocytosis and phagocytosis

Billions of cells naturally die every day in healthy individuals. These cells are being phagocytosed and cleared by macrophages in a process termed efferocytosis. Since inflammation drives cell death, which in turn exacerbates inflammation, efferocytosis is critical for inflammation resolution. Macrophages recognize apoptotic cells *via* ligand-receptor interactions, followed by engulfment of the dead cells or debris [reviewed in (Thorp and Tabas, 2009)]. This induces phagosome-lysosome fusion and degradation of the engulfed contents. A defective efferocytosis pathway is one of the hallmarks of chronic inflammatory diseases, resulting in accumulation of dead cells, which enhances inflammation and creates a feed-forward loop of inflammation and cell death. Efferocytosis is a quieter form of phagocytosis; while phagocytosis is followed by antigen presentation and T cell activation, in efferocytosis, internalization of apoptotic cells leads to its sequestration towards endosomes, taking it away from MHCII- mediated antigen presentation (Yin and Heit, 2021).

## Adipose tissue inflammation and insulin resistance

Diabetes, one of the major public health challenges worldwide, is estimated to be affecting 537 million people between ages 20–79. This number is estimated to increase to 784 million by 2045 (Federation, 2021). The increase in the prevalence of type 2 diabetes is not surprising, since obesity is one of its major causes (Dale et al., 2017). Apart from the economic burden posed by diabetes, it is also a major driver of mortality. In 2021 alone, ∼6.7 million adults died as a result of diabetes complications, without accounting for COVID-19-related mortality that is exacerbated by diabetes (Federation, 2021).

The main role of the adipose tissue (AT) is to store excess nutrients, mostly in the form of triacylglycerols, and release these lipids in times of energetic needs, e.g., exercise, under-nutrition etc. [reviewed in (Heffron et al., 2020)]. Obesity induces extensive remodeling of the AT, including tissue expansion and changes in structure and cellular composition. AT expansion is caused by adipocyte hyperplasia (cell number increase) and hypertrophy (cell enlargement) to allow the storage of excess lipids (Berry et al., 2014). The inability to appropriately expand AT in obesity leads to ectopic lipid deposition in the liver and skeletal muscle and may be an underlying cause of insulin resistance. These processes also cause dramatic changes to the immune milieu in the adipose (Jaitin et al., 2019; Weinstock et al., 2019). Macrophages play a critical role in lipid storage and utilization in the AT, tightly regulating metabolic health [reviewed in (Peterson et al., 2018)]. There is an active debate regarding the role of AT macrophages (ATMs), with early studies suggesting they promote metabolic pathologies such as insulin resistance. However, the common modern view is that ATMs are multifunctional, with some having detrimental and some beneficial effects on metabolic inflammation (Table 1) (Coats et al., 2017; Weinstock et al., 2020).

**TABLE 1:** Summary of factors in macrophages that protect from (green) or promote (red) adipose tissue inflammation and insulin resistance.

	Factor	Function	References
**Lipid handling**	PPARγ	Regulates metabolic processes such as lipid production, storage and metabolism. Promotes pro-resolving polarization and efferocytosis	Luo et al. (2019), Montaigne et al. (2021)
	TREM2	Marker for foam cell like macrophages, involved in phagocytosis and lipid catabolism	Kober and Brett (2017), Jaitin et al. (2019)
	LPL	Uptake and metabolism of cholesterol and fatty acids from lipoproteins, as well as the synthesis of cholesterol esters and triglycerides	Mead et al. (2002), Aouadi et al. (2014)
**Cytokine**	IL-1β	Reprograms adipocyte metabolism, promotes myelopoiesis and adipocyte insulin resistance	Larsen et al. (2007), Vandanmagsar et al. (2011), Gao et al. (2014), Nagareddy et al. (2014), Zhou et al. (2020)
	IL-13	inhibit inflammatory activation of ATMs and improve glucose and insulin tolerance	Kwon et al. (2014)
	IL-33	induces the production of IL-5, IL-6 and IL-13 which can exert protection against insulin resistance, also reduces obesity	Miller et al. (2010)
	IL-10	Suppress the production of pro-inflammatory cytokines and increases sensitivity to insulin	Lumeng et al. (2007), Hong et al. (2009)
**Lipid mediators**	COX-2	Inhibits accumulation of fat in ATMs and their local proliferation, as well as monocyte recruitment	Pan et al. (2022)
	BLT1	Promotes trafficking of monocyte to AT and increases hepatic triglyceride levels	Spite et al. (2011)
	RvD1, RvE1	Improve hyperglycemia and hyperinsulinemia. RvD1 promotes reparative macrophage polarization	Hellmann et al. (2011), Pal et al. (2020)
**miRNAs**	miR-155	Inhibits insulin signaling through suppression of *Pparg*	Tryggestad et al. (2019)
	miR-17–92	Upregulates *Il10* and suppresses *Tnfa*	Zhang et al. (2020)
	miR-30	Reduces inflammatory polarization, including TNFa and CCL2 production	Miranda et al. (2018)
**Efferocytosis/phagocytosis**	MERTK	Crucial role in the recognition and subsequent ingestion of apoptotic cells	Suresh Babu et al. (2016)
	ADAM9	Cleaves and inactivates Mertk	Suresh Babu et al. (2016)
	EPO	Elevates PPARγ levels	Luo et al. (2019)

### Macrophage origin

Several studies have investigated the origin of AT macrophages in obesity. In a seminal study, Weisberg et al. found that ATMs are derived from bone marrow progenitors (Weisberg et al., 2003). By performing bone marrow transplantation, they showed that 85% of ATMs were derived from the donor bone marrow 6 weeks post-transplantation. This ATM accumulation preceded the development of insulin resistance, and was proposed to be the causal link between obesity and insulin resistance (Xu et al., 2003).

Two studies discovered that increased levels of chemokine (C-C motif) ligand 2 (CCL2) in AT and plasma is responsible for increased macrophage accumulation in obesity, thus suggesting monocyte recruitment to the AT. They found that there was upregulation of CCL2 in the adipose tissue and plasma of genetically obese (db/db) and high-fat diet (HFD)-fed mice. In both studies, a transgenic mouse overexpressing CCL2 was created, which was sufficient to induce macrophage infiltration into AT, insulin resistance, and increase hepatic triglyceride content, even with low-fat diet feeding (Kamei et al., 2006; Kanda et al., 2006). On the other hand, CCL2 global deficiency reduced ATM accumulation and improved insulin intolerance and hepatic steatosis associated with adiposity in diet-induced obese (DIO) mice (Kanda et al., 2006). Along the same lines, C-C chemokine receptor type 2 (CCR2), the receptor for CCL2, CCL7 and CCL8, is also involved in the development of metabolic dysfunction and ATM recruitment. In fact, obesity causes dramatic upregulation of CCL2, CCL7 and CCL8 in the AT (Weisberg et al., 2006). Knockout of *Ccr2* in DIO mice shows lower fasting blood glucose and improved glucose and insulin tolerance (Weisberg et al., 2006). Subsequently, others have also showed the importance of CCR2 in the development of metabolic dysfunction and ATM accumulation in obese mice (Ito et al., 2008; Sullivan et al., 2013). Contrarily, a recent study found that resident ATMs do not express CCR2, but rather Timd4, derived from early embryonic tissues. These resident ATMs produce platelet-derived growth factor (PDGF) cc that regulates lipid storage in adipocytes. HFD increases *Pdgfc* expression in ATMs, and its blockade prevents adipocyte hypertrophy, possibly by downregulating adipogenesis (Cox et al., 2021). Further studies, using cell depletion, show that resident ATMs are integral to metabolic health (Chen et al., 2021). CD169, which is presumably expressed more on non-recruited ATMs was used to deplete this population, without affecting monocyte-derived ATMs. Absence of CD169^+^ macrophages increased AT mass following HFD and promoted adipocyte hypertrophy. CD169^+^ ATMs were proposed to maintain the AT vasculature integrity (Chen et al., 2021). An earlier study elegantly described ATMs that are in close contact with vasculature and were termed vascular-associated macrophages (Silva et al., 2019). These cells were shown to protect the AT with their high endocytic capacity, thus creating a buffer from the circulation and preventing the entrance of insults to the AT. Obesity impairs the endocytosis ability of vascular-associated macrophages, allowing more promiscuous entry of substances from the blood to the AT (Silva et al., 2019).

HFD does not only increase total ATM numbers but also attracts a unique ATM subpopulation that is minimally present in AT under steady state conditions. The obesity-driven infiltrating ATMs are bone marrow-derived, become lipid-laden (foam cells) and express markers such as *Cd11c*, *Cd9*, and *Trem2*. The majority of these infiltrating ATMs form classical crown-like structures around dead or damaged hypertrophic adipocytes. CD11c^+^ ATMs in obese mice were shown to possess high lipid metabolism and bioenergetic activity. CD11c^+^ ATMs are equipped with specific receptors (e.g., *Cd36*, *Msr1*) and enzymes (e.g., *Lpl*, *Fabp4*, and *Mgl1*) to take up and process lipids. Altogether it seems that monocyte-derived and resident ATMs have distinct roles in regulating AT functions, which requires further elucidation.

### Lipid handling

Lipid handling is important in macrophages to maintain metabolic health. This was suggested in an early study that investigated the role of PPAR-γ in macrophages. PPAR-γ is a transcription factor and master regulator of metabolic processes, including lipid production, storage and metabolism [reviewed in (Montaigne et al., 2021)]. Macrophage-specific knockout of *Pparg* results in glucose intolerance systemically, as well as in the liver and muscle, even in lean conditions (Hevener et al., 2007). In the AT, macrophage *Pparg* knockout impaired the expression of numerous genes involved in lipid uptake, synthesis and β–oxidation. Furthermore, adipocytes co-cultured with *Pparg* ^−/−^ ATMs show inhibition of insulin-stimulated glucose uptake, directly showing causality of macrophage *Pparg* deficiency and adipose insulin resistance (Odegaard et al., 2007). *Pparg* deficiency further prevents the beneficial effects of the anti-diabetic drug Rosiglitazone in obese mice (Hevener et al., 2007).

*Trem2* is a hallmark gene for macrophages that accumulate in adipose and other lipid-rich tissues (Kober and Brett, 2017). It is a receptor for multiple ligands, including lipoproteins and phospholipids, and is a major driver of tissue-level immune cell remodeling [reviewed in (Kober and Brett, 2017)]. *Trem2* is expressed on a subset of ATMs, which were termed lipid-associated macrophages (LAM). LAMs arise from circulating monocytes and are positioned around enlarged adipocytes. Functionally, LAMs are thought to be involved in phagocytosis and lipid catabolism, through a TREM2-induced transcriptional program. Consequently, *Trem2* deficiency prevents the formation of crown-like structures in the obese AT, causing massive adipocyte hypertrophy, systemic hypercholesterolemia, inflammation, and glucose intolerance (Jaitin et al., 2019). Hence, *Trem2* expression limits obesity-induced metabolic dysfunction, possibly by promoting the removal of dysfunctional defective adipocytes by ATMs. Moreover, macrophage accumulation around large adipocytes in obese adipose might be driven by TREM2 to locally contain toxic lipids and other inflammatory mediators from harming their surroundings.

Lipid uptake by ATMs was also shown to modulate systemic glucose tolerance and promote inflammation and metabolic derangement in obesity. For instance, lipoprotein lipase (LPL) is an enzyme that hydrolyzes triglycerides and phospholipids for lipid uptake, which can be used as energy or stored in the form of triglycerides in lipid droplets. LPL is involved in the uptake and degradation of cholesterol and fatty acids from lipoproteins, as well as the synthesis of cholesterol esters and triglycerides (Mead et al., 2002) and its role was extensively investigated in macrophages. In one study, *Lpl* was knocked down specifically in ATMs using nanoparticles containing siRNA against this gene. *Lpl* knockdown decreased foam cell formation in visceral AT of obese mice and reduced fatty acid uptake from very low-density lipoprotein (VLDL) hydrolysis. *Lpl* knockdown in ATMs decreased the expression of genes involved in fatty acid uptake and esterification (*Cd36* and *Dgat2*, respectively), resulting in higher levels of circulating free fatty acids and glucose intolerance in obese mice (Aouadi et al., 2014). That said, more recent studies did not find a role for macrophage LPL in obesity-related morbidity. In this study, the authors compared the effects of genetic global or macrophage-specific deletion of *Lpl* in obesity, peritonitis and atherosclerosis regression. They demonstrated that ATMs accumulated less fat only in the global, but not macrophage-conditional knockouts, which was associated with a more pro-reparative macrophage phenotype (Chang et al., 2019). *In vitro*, macrophage *Lpl* is needed for lipid uptake from VLDL (Chang et al., 2019).

Augmented VLDL-VLDLR signaling was shown in obese ATMs to aggravate AT inflammation and insulin resistance. The presence of VLDLR in macrophages from obese mice increased intracellular level of triglyceride, which stimulates the expression of pro-inflammatory genes, such as *Nos2, Tnfa, Ccl2*, serum amyloid A, *Il1b* and *Ifng*. It was further found that the levels of C16:0 ceramides are decreased in VLDLR deficient macrophages, which are less inflammatory, through the reduced activity of mitogen-activated protein kinases (MAPK) (Kober and Brett, 2017).

ATMs also have an important role in lipid trafficking, independent of their inflammatory phenotype. The expansion of AT during obesity induces a program of lysosome biogenesis in ATMs that is associated with lipid catabolism. This program is induced by factors produced by adipocytes and tightly coupled to lipid accumulation in ATMs. Inhibition of ATM lysosomal function impairs lipid metabolism, increases lipid content in ATMs and reduces AT lipolysis, contributing to worsening of metabolic syndrome (Weisberg et al., 2003). Contrarily, others have shown that lipolysis and lipid storage in macrophages do not affect adipose tissue inflammation. In a recent study, Xanthe et al. demonstrated that hypoxia inducible lipid droplet associated (HILPDA), a protein that promotes lipid storage in macrophages, was dispensable in obesity-induced AT inflammation. They showed that myeloid *Hilpda* deficiency markedly reduces intracellular lipid levels in macrophages *in vitro* and in the AT, without affecting inflammation and glucose intolerance (Xu et al., 2003). The discrepancy between these reports might be due to the importance of macrophage lipid uptake and metabolism, rather than storage. Moreover, it is unclear how *Hilpda* or *Lpl* deficiency alter the types of lipids utilized by macrophages, which can also greatly influence their phenotype and outcomes of obesity.

### Cytokines

Numerous classical cytokines play a critical role in adipose tissue inflammation and diabetes. Several seminal papers described the involvement of pro-inflammatory cytokines, namely, IL-1β, in the pathogenesis of obesity and diabetes. Importantly, it was shown in a clinical trial that Anakinra, an IL-1 receptor antagonist, improves systemic inflammation and glycemia, with no effect on insulin sensitivity in type II diabetes patients (Larsen et al., 2007). NLR family pyrin domain containing 3 (NLRP3) is the core component of the inflammasome, responsible for IL-1β processing and secretion. Both *Il1b* and *Nlrp3* gene expression is increased in the obese AT of mice and humans, which is reversible by weight loss (Vandanmagsar et al., 2011). Interestingly, *Nlrp3* ^*−/−*^ mice fed HFD are more insulin and glucose sensitive, and protected from hepatic steatosis, compared with *Nlrp3* sufficient controls (Vandanmagsar et al., 2011). Mechanistically, Nagareddy et al. (2014) showed that ATMs are stimulated by S100A8/A9 that are produced by obese adipocytes. In response, ATMs secrete IL-1β, which activates immune progenitors in the bone marrow to produce more monocytes and neutrophils, thus inducing a feed-forward loop of AT inflammation. IL-1 was further demonstrated to reprogram adipocyte metabolism, exacerbating adipose dysfunction and obesity (Zhou et al., 2020). Others attempted to link insulin resistance itself to macrophage-derived IL-1β. One such study investigated how IL-1β-primed macrophages influence adipocyte insulin signaling *in vitro*. They found that macrophages treated with IL-1β induce adipocytic expression of pro-inflammatory cytokines (i.e., *Ccl2*, *Il6* and *Il8*) and blunt insulin signaling. These effects were largely reversed with IL-1β antagonism (Gao et al., 2014).

An important cytokine induced by IL-1β is IL-6, which is, thus, widely considered pro-inflammatory. However, some evidence indicate that IL-6 is necessary for metabolic health. Some of those studies show that IL-6 deficiency led to development of obesity, liver inflammation and insulin resistance even during low-fat feeding (Wallenius et al., 2002; Matthews et al., 2010). Experimental evidence show that IL-6 produced by adipocytes is deleterious, while myeloid and muscle cells production of IL-6 is protective in obesity, possibly explaining the discrepancy between its inflammatory nature and metabolic importance (Han et al., 2020).

Early reports showed that macrophage TNFα contributes to insulin resistance and its deletion alleviates insulin resistance (Stephens and Pekala, 1991; Hotamisligil et al., 1993; De Taeye et al., 2007). However, it was recently reported that the deletion of macrophage TNFα did not influence insulin resistance and hepatic lipid accumulation in obese mice, indicating that macrophages are not the source of TNFα that causes metabolic dysfunction (Aladhami et al., 2021).

Although macrophages have been found to produce pro-inflammatory cytokines that enhance insulin resistance, experimental evidence demonstrate that macrophages also secrete and respond to anti-inflammatory cytokines, enhancing insulin sensitivity. For example, IL-13 produced by obese adipocytes was shown to inhibit inflammatory activation of ATMs and improve glucose and insulin tolerance (Kwon et al., 2014). In addition, IL-33 treatment of adipocytes and ATMs induces the production of IL-5, IL-6 and IL-13 which can exert protection against insulin resistance. Furthermore, IL-33 administration reduces adiposity, while also attenuating insulin and glucose intolerance caused by obesity (Miller et al., 2010). IL-10 has also been found to suppress the production of pro-inflammatory cytokines and protect from TNFα-induced insulin resistance in obesity (Lumeng et al., 2007). Moreover, IL-10 overexpression increases insulin sensitivity, protects skeletal muscle from obesity-associated macrophage infiltration and decreases production of inflammatory cytokines (Hong et al., 2009).

### Lipid mediators

Plasma and adipose tissue concentration of SPMs, leukotrienes and prostaglandin are markedly changed in human obesity and diabetes, compared to lean healthy conditions (Claria et al., 2013; Titos et al., 2016; Schulte et al., 2020). Chronic inflammation in obesity is a consequence of the failure to actively resolve inflammation, possibly due to reduction in lipid mediators. For instance, HFD increases palmitic acid, the major free fatty acid released by adipocytes, causing upregulated expression of *Ptgs2* in macrophages. *Ptgs2* knockout in myeloid cells of DIO mice leads to accumulation of fat in ATMs, an increase in monocyte recruitment and local proliferation of ATMs, as well as insulin resistance, inflammation and AT fibrosis. These data were recapitulated when myeloid cells did not express the PGE_2_ receptor EP4 (Pan et al., 2022). Conversely, Hellmann et al. (2013), found that in db/db and DIO mice, palmitic acid stimulates COX-2 and increases prostaglandin production, leading to accumulation of neutrophils and impair macrophage phagocytosis in a model of peritonitis. COX-2 or EP2 (prostaglandin 2 receptor) inhibition decreased neutrophil and apoptotic cell accumulation in the peritoneum of db/db mice, suggesting that COX-2/PGE_2_ impair inflammation resolution in obese mice. Taking these two studies into account, it seems that PGE_2_ is important in macrophages to promote inflammation resolution in obesity. However, PGE_2_ might be dominantly deleterious when activating other, yet unknown, cells.

The role of BLT-1 (leukotriene B4 receptor) in promoting trafficking of monocyte to adipose tissue and chronic inflammation in obesity has also been elucidated. HFD increases the circulating levels of monocytes that express *Blt1*, while its knockdown prevents the accumulation of ATMs and expression of *Il6* and *Ccl2*. *Blt1* deficiency was also associated with decreased hepatic triglycerides, leading to improved systemic glucose and insulin tolerance. This indicates that BLT-1 may contribute to obesity-induced metabolic derangement (Spite et al., 2011).

Resolvins were also shown to improve insulin and glucose tolerance. In obese mice, supplementation with either RvD1 (Hellmann et al., 2011) or RvE1 (Pal et al., 2020) improved fasting glucose and insulin levels. RvD1 treatment was further shown to improve AT inflammation and promote reparative macrophage polarization (Hellmann et al., 2011), suggesting that these resolvins might be considered as treatment for obesity-induced insulin resistance.

### MicroRNAs

ATMs from obese animals secrete exosomes containing miRNAs that are taken up by insulin target cells, both *in vitro* and *in vivo*, which leads to cellular and systemic insulin resistance and glucose intolerance. Among these is miR-155, which inhibits insulin signaling and glucose tolerance through suppression of *Pparg* (Tryggestad et al., 2019). In contrast, treatment of obese mice with ATM-exosomes derived from lean mice leads to improvement in glucose tolerance and insulin sensitivity, indicating that ATM-exosomes perform both detrimental and beneficial actions depending on the metabolic state of the AT (Ying et al., 2017; Gao et al., 2021). Another miR that is packaged in exosomes and can regulate metabolic health is miR-690. It has been shown that IL-4/13 treated bone marrow-derived macrophages produce miR-690 rich exosomes. Administration of these exosomes to obese mice improves their glucose and insulin intolerance. A similar protective phenotype was observed after transferring exosomes that exclusively carry miR-690, and no other miR, to obese mice (Ying et al., 2021).

In addition to miR-690 there are other miRs that promote metabolic health. PPARγ, which is important for macrophage anti-inflammatory polarization, is responsible for the expression of miR-223. Deficiency of miR-223 in HFD-fed mice causes marked glucose intolerance and insulin resistance (Zhuang et al., 2012; Ying et al., 2015). In addition, miR-223 reduces Pknox1 levels, a protein that induces pro-inflammatory macrophage polarization (Zhuang et al., 2012). miR-17–92 family are also crucial regulators of the balance between pro-inflammatory and anti-inflammatory cytokines. HFD fed miR-17–92^−/−^ mice had increased body weight, adiposity and TNFα but reduced IL-10 when compared with wild type mice. Treatment of miR-17–92^−/−^ mice with anti-TNFα suppresses obesity and increases IL-10 *via* upregulation of Fos Proto-Oncogene expression, suggesting that miR-17–92 controls obesity by upregulating *Il10* and suppressing *Tnfa* (Zhang et al., 2020).

In addition, HFD downregulates miR-30 expression and subsequently upregulates pro-inflammatory responses in ATMs. Enhancement of miR-30 in RAW264.7 (macrophage cell line) reduced macrophage inflammatory polarization and decreased TNFα and CCL2 production by inhibiting the Notch ligand DLL4. This suggests that enhancement of miR-30 may produce an anti-inflammatory effect and can be considered for resolution of inflammation in obesity (Miranda et al., 2018).

### Efferocytosis and phagocytosis

Efferocytosis is essential for successful resolution of inflammation and maintenance of tissue homeostasis. Obesity and its associated complications compromise the function of macrophages leading to prolonged and impaired resolution of inflammation. Several factors have been found to be responsible for impaired efferocytosis and phagocytosis in obesity and diabetes. c-Mer tyrosine kinase (MerTK) has been shown to play a crucial role in the recognition and subsequent ingestion of apoptotic cells by macrophages. Human diabetic hearts were shown to have reduced levels of MerTK, coupled with a decrease in miR-126. Mechanistically, hyperglycemia reduces macrophage miR-126, which cannot inhibit *Adam9* expression. ADAM9 cleaves and inactivates MerTK, thereby inhibiting efferocytosis (Suresh Babu et al., 2016). Another cause of impaired efferocytosis and phagocytosis in obesity and diabetes is the altered ratio between free fatty acids and pro-resolving lipids. Ob/ob mice were shown to have more palmitic and stearic acid, and less EPA and DHA (Li et al., 2009). Interestingly, treatment of naïve macrophages with saturated fatty acids greatly impairs their efferocytic capability. *In vivo*, ob/ob atherogenic mice had more apoptotic cells in their plaques compared to atherogenic mice that are not on the ob/ob background, which was reversed by feeding an omega-3 rich diet (Li et al., 2009).

In addition to the effects of prostaglandins on impairment of efferocytosis in obesity [discussed above (Hellmann et al., 2013)], it was shown that obesity reduces macrophage erythropoietin (EPO), which elevates PPARγ levels, required for the macrophage pro-reparative phenotype. In a peritonitis model, treatment of obese mice with recombinant human EPO abrogates the defects in efferocytosis caused by obesity and stimulates inflammation resolution (Luo et al., 2019).

Weight loss in obesity might also improve phagocytic capabilities of ATMs. Single-Cell RNA sequencing of lean, obese and calorically restricted visceral AT immune cells has revealed a new distinct subpopulation of ATMs that is unique to weight loss. These ATMs express many phagocytosis/efferocytosis-related genes, such as *Fcgr4*, *Axl*, *Mertk* and *Fcer1g*. It was further demonstrated that caloric restriction-induced weight loss promotes the appearance of multinucleated ATMs, further supporting their increased phagocytic capacity. However, the function of these newly found ATMs remains to be understood (Weinstock et al., 2019). Although this study suggests that weight loss promotes beneficial changes to ATMs, two recent papers demonstrate that weight loss induced by switching from high-to low-fat diet does not reverse the obesity-induced inflammatory phenotype of ATMs (Caslin et al., 2022; Hata et al., 2023). Both these studies show that formerly obese ATMs produce more inflammatory cytokines than lean ones, and in comparable amounts to obese ATMs upon stimulation with lipopolysaccharide (LPS). These data suggest that obesity has long-lasting effects on the inflammatory state of macrophages, which can further exacerbate obesity comorbidities.

Another recent report elegantly characterized the immune compartment in obese mice following diet switch-induced weight loss and regain. Cottam et al. made several interesting observations. First, they did not find similar weight loss specific, *Fcgr4* expressing ATMs, which might be due to several differences in the design of both studies. Some differences include changes in diet composition during weight loss (from 60% high fat diet to 10% low fat diet) and the duration of weight loss (9 weeks), while Weinstock et al. maintain the same HFD throughout but reduce food amounts by 30% for 2 weeks to allow weight loss. Second, they observed an ATM subpopulation that cycles together with weight, accumulating with obesity, disappearing in weight loss and reappearing in greater amounts with weight regain. Some of their marker genes include *Stmn1, Pclaf, Saa3 and Slpi,* suggesting they are proliferative and might be involved in efferocytosis, however, not much is known about these cells otherwise. It remains to be seen if these cycling cells contribute to the enhanced inflammation occurring with weight regain. Third, the data indicate that many transcriptional changes induced by obesity in resident ATMs and LAMs are not recovered upon weight loss, possibly priming them to respond more robustly upon weight regain (Cottam et al., 2022). How weight loss and regain influence the immune microenvironment in the AT is an important question that will require further investigations.

## Atherosclerosis

Cardiovascular diseases (CVDs) are the leading cause of health concern globally, accounting for about one-third of all deaths. Atherosclerosis, one of the most dangerous CVDs, is characterized by the formation of plaques in the arteries, which ultimately leads to various forms of disease manifestation, depending on the plaque location, including coronary artery, peripheral artery and cerebrovascular disease, frequently resulting in what is commonly referred to as heart attack and stroke. The disease is initiated by the accumulation of modified-cholesterol moieties under the endothelial layer (Witztum and Steinberg, 1991), which induces the onset of a vicious inflammatory cycle. Stressed endothelial cells in the arteries respond by increasing their cell surface levels of various leukocyte adhesion molecules, such as Intercellular adhesion molecule (ICAM) - 1 (Kevil et al., 2001) and Vascular Cell Adhesion Molecule 1 (VCAM-1) (Hellmann et al., 2011), while they and other cells secrete chemokines that direct monocytes to the site of inflammation. Monocytes then adhere to endothelial ICAM-1 and VCAM-1 and transmigrate to penetrate the arterial wall. Once resident in the artery, monocytes become macrophages, working to engulf trapped LDL molecules through the upregulation of their scavenger receptors (Libby, 2002).

Due to the inflammatory environment, cells may undergo apoptosis. The failure to clear these dying cells by macrophages leads to secondary cellular necrosis which, over time, develops into the necrotic core, the hallmark of plaque instability. The necrotic core is also a major source of danger-associated molecular patterns, matrix metalloproteases and pro-inflammatory cytokines, which further exacerbate the disease and may ultimately lead to plaque rupture. The modern view of the role of macrophages in atherosclerosis came from pioneering studies that demonstrated macrophage presence in atherosclerotic plaques (Cookson, 1971) and the role of monocyte-derived macrophages recruitment in plaque progression (Swirski et al., 2007). With the advancements of single-cell RNA-sequencing, we have further clarity on the different plaque macrophage subtypes. Meta-analysis of plaque leukocytes described 5 major subtypes of macrophages (Cochain et al., 2018; Kim et al., 2018; Winkels et al., 2018; Lin et al., 2019; Zernecke et al., 2020). Since single-cell RNA-sequencing is continuously being collected, even more macrophage subtypes are being discovered in mice and humans (Fernandez et al., 2019; Pan et al., 2020; Eberhardt and Giannarelli, 2022), however, their distinct functions are only now beginning to be elucidated (Table 2).

**TABLE 2:** Summary of factors in macrophages that protect from (green) or promote (red) atherosclerosis.

	Factor	Function	References
**Lipid handling**	ABCA1, ABCG1, ABCA5	Reduce cellular cholesterol load *via* reverse cholesterol transport	Terasaka et al. (2007), Out et al. (2008), Ray et al. (2020)
	LXR	Upregulates cholesterol transport genes, thus improves cholesterol efflux	Joseph et al. (2002), Tangirala et al. (2002), Levin et al. (2005), Teupser et al. (2008), Feig et al. (2010)
	PPARγ	Increases the uptake and degradation of cholesterol and fatty acids. Promotes inflammation resolution, IL-10 production and prevents TNFa and IL-1β synthesis	Babaev et al. (2005), Choudhury et al. (2005), Majai et al. (2007), Heming et al. (2018)
	TREM2	Senses extracellular lipids and suppresses proinflammatory cytokine production	Turnbull et al. (2006), Jaitin et al. (2019), Zernecke et al. (2020)
**Cytokine**	TNFa	Induces scavenger receptor expression on macrophages which promotes foam cell formation	Argmann et al. (2001), Michael et al. (2012)
	IL-1β	Induces scavenger receptor expression on macrophages and foam cell formation. Pro-inflammatory	Argmann et al. (2001), Michael et al. (2012)
	TGF-β	Inhibits expression and secretion of pro-inflammatory cytokines. Reduces E-selectin and VCAM1 to reduce monocyte recruitment	Gamble et al. (1995), DiChiara et al. (2000), Argmann et al. (2001), Michael et al. (2012)
	IL-10	Inhibits leukocyte-endothelium interaction, plaque progression, NF-κB activation, metalloproteinase production and cell death. Promotes efferocytosis	Lacraz et al. (1995), Wang et al. (1995), Lentsch et al. (1997), Downing et al. (1998), Mallat et al. (1999a), Mallat et al. (1999b), Henke et al. (2000), Caligiuri et al. (2003)
	IL-33	Reduces foam cell formation, induces IL-4, IL-13 and upregulates cholesterol efflux genes	Miller et al. (2008), McLaren et al. (2010)
	IL-4	Promotes expression of *Arg1*, thus stimulating continuous efferocytosis	Weinstock et al. (2021)
	IL-13	Pro-resolving, anti-inflammatory	Cardilo-Reis et al. (2012)
**Lipid mediators**	COX1	Promotes atherosclerosis development	Pratico et al. (2001)
	MaR1, RvD1 and RvD2	Inhibit macrophage accumulation. Stimulate efferocytosis and release of calreticulin, thus decreasing necrotic cores	Fredman et al. (2016), Viola et al. (2016)
**miRNAs**	miR-33	Suppresses the expression of lipid metabolism genes and efferocytosis	Gerin et al. (2010), Ouimet et al. (2017), Afonso et al. (2021)
	miR-144	Reduces RCT by downregulating ABCA1	Hu et al. (2014)
	miR-342-5p	Promotes the expression of pro-inflammatory genes through inhibition of AKT1 and subsequent induction of miR-155	Wei et al. (2013)
**Efferocytosis/phagocytosis**	CD47	Protects apoptotic cells from being efferocytosed	Oldenborg et al. (2000), Gardai et al. (2005), Schrijvers et al. (2005), Kojima et al. (2016)
	Calreticulin	Marks cells for clearance	Fabbrini et al. (2010)
	MERTK	Identifies apoptotic cells and initiates efferocytosis	Thorp et al. (2008), Cai et al. (2017)
	ARG1	Promotes uptake of multiple apoptotic cells	Yurdagul et al. (2020)

### Macrophage origin

Macrophages reside in the healthy aorta (Jongstra-Bilen et al., 2006) and were thought to be derived from both embryonic and monocytic origins (Ensan et al., 2016). However, it was recently demonstrated that unlike most tissue resident macrophages, aortic macrophages are not derived from embryonic tissues (i.e., yolk sac and fetal liver), but rather from circulating monocytes recruited to the aorta shortly after birth (Williams et al., 2020). These cells were named Mac^AIR^ and were found to proliferate locally to maintain their population. In hypercholesterolemic conditions, Mac^AIR^ form the first foam cells. Mac^AIR^ were further shown to induce the initial recruitment of monocytes to the aorta and their depletion delayed atherosclerosis initiation (Williams et al., 2020). Meta-analysis of plaque leukocytes identified lymphatic vessel endothelial hyaluronan receptor-1 (Lyve-1) as a marker for resident macrophages (Zernecke et al., 2020).

Incoming monocytes are the source of most plaque macrophages (Williams et al., 2020). Early studies implicated Ly6C^hi^ monocytes in forming inflammatory macrophages within lesions (Swirski et al., 2007). Additionally, using knockout mouse models, several chemokines and their receptors (such as CCR2-CCL2 (Boring et al., 1998; Gu et al., 1998), CCR5-CCL5 (Veillard et al., 2004; Braunersreuther et al., 2007) and CX_3_CR1-CX_3_CL1 (Lesnik et al., 2003)] were discovered to play a major role in monocyte recruitment into the atheroma. CCR2 and CX_3_CR1 were shown to induce the recruitment of Ly6C^hi^, while CCR5 is responsible for Ly6C^lo^ monocyte recruitment (Tacke et al., 2007). Importantly, blocking monocyte recruitment in mice *via* a combination of *Ccl2* and *Cx3cr1* double knockout with CCR5 inhibition severely impaired disease progression and reduced plaque size by 90% (Combadiere et al., 2008). Monocyte-derived macrophages were also shown to persist locally through proliferation in plaques (Robbins et al., 2013). These results raised the hope for a simple anti-atherosclerotic intervention in the form of inhibition of monocyte recruitment. However, this is more complicated than expected, due to the dichotomous role of macrophages in causing and resolving inflammation. Of importance, resolution of atherosclerosis was completely abrogated in CCR2, but not CCR5-deficient mice (Rahman et al., 2017). This suggests that while serving as instigators of atherosclerosis development and progression, monocytes and in particular the Ly6C^hi^ subset, are indispensable for atherosclerosis resolution.

Another interesting source of cells that are perceived as macrophages in atherosclerotic plaques is vascular smooth muscle cells (SMCs). In hypercholesterolemic conditions, SMCs may show features of transdifferentiating to macrophage, downregulating SMC-specific markers and increasing their expression of macrophage markers (Andreeva et al., 1997; Rong et al., 2003; Mulvihill et al., 2004; Allahverdian et al., 2014; Shankman et al., 2015) (reviewed in (Doran et al., 2008; Orr et al., 2010; Bennett et al., 2016)]. Their function is not completely understood yet, and they will not be expanded on further in this review.

### Lipid handling

Another critical role of macrophages in the atheroma is to assist in the clearance of excess cholesterol and deliver it to the liver for redistribution, breakdown or excretion. This process is termed reverse cholesterol transport (RCT). Apolipoprotein A1, the signature protein component of HDL, interacts with cholesterol transporters such as ABCA1 (Out et al., 2008), ABCG1 (Terasaka et al., 2007) and ABCA5 (Ray et al., 2020) in various cell types (including macrophages) to facilitate the withdrawal of excess cellular cholesterol. Therefore, HDL acts as both the receiver and transporter of excess cholesterol from peripheral tissues into the liver. Additionally, HDL possesses antioxidants and anti-inflammatory potential that can aid plaque regression [reviewed in (Tall et al., 2008)]. Multiple studies have tried increasing plasma HDL levels to reduce atherosclerosis with mixed results in preclinical models and disappointing results in clinical trials. This might be because enhancement of RCT requires a coordinated response between cholesterol efflux, the capability of HDL to accept cholesterol and finally the ability of liver cells to accept it for downstream processing. Further understanding of HDL and its local functions is needed to maximize its therapeutic benefits.

RCT is induced by liver X receptors (LXRs), which upregulate the expression of various cholesterol transport genes in response to a lipid-rich environment. Transplantation of bone marrow lacking LXRα and β into *ApoE* ^*−/−*^ and *Ldlr* ^*−/−*^ mice results in aberrant regulation of cholesterol transporter genes, lipid accumulation in macrophages and increased atherosclerosis, phenotypically mimicking Tangier disease in humans (Tangirala et al., 2002). Similarly, macrophage overexpression of LXRα in *Ldlr* ^*−/−*^ mice results in reduction in lesion area at the brachiocephalic artery, while cholesterol efflux is significantly elevated (Teupser et al., 2008). Administration of non-steroidal LXR agonist (GW3965) display potent anti-atherogenic activity by inducing the expression of *Abca1* and *Abcg1* in *Ldlr* ^*−/−*^ mice (Joseph et al., 2002). Moreover, administration of the LXR agonist T0901317 to mice harboring existing plaques resulted in plaque regression and remodeling towards a stable phenotype. T0901317 failed to exert its anti-atherogenic effects in the absence of macrophage LXR expression (Levin et al., 2005). Furthermore, LXR is required for atherosclerosis regression since it induces CCR7-mediated egress of macrophages and dendritic cells from plaques (Feig et al., 2010). LXR further inhibits inflammatory responses by inducing macrophage phagocytic function and inhibiting macrophage proliferation (144). The loss of myeloid LXR reduces the expression of *Trem2* and its related genes, thus promoting atherosclerosis progression (Endo-Umeda et al., 2022). Altogether, these studies indicate that LXR activation in macrophages has a protective effect. However, LXR can lead to hepatic fat deposition and inflammation, causing a drawback to its potential clinical use [reviewed in (Fessler, 2018)].

Another key regulator of macrophage lipid metabolism and inflammation is PPARγ. Activation of PPARγ in macrophages leads to the expression of genes involved in the uptake and degradation of cholesterol and fatty acids, as well as the synthesis of anti-inflammatory molecules such as IL-10 upon stimulation with LPS (Majai et al., 2007; Heming et al., 2018). This stimulates resolution of inflammation and prevents foam cell formation, which is a key step in the development of atherosclerotic plaques (Choudhury et al., 2005). In contrast, inhibition of PPARγ in macrophages leads to the expression of pro-inflammatory genes and the synthesis of pro-inflammatory molecules such as TNF-α and IL-1β. This promotes inflammation and foam cell formation, which contributes to the development of atherosclerotic plaques. Macrophage specific knockout of *Pparg* results in significantly larger plaques with more macrophages in comparison to *Pparg* sufficient controls. This was accompanied by increased macrophage recruitment and *Ccr2* overexpression, while *in vitro* assays revealed reduced uptake of oxidized but not acetylated LDL (Babaev et al., 2005).

Another key regulator of lipids in macrophages is LPL. Elucidation of the role of LPL in atherosclerosis was challenging at first because homozygous knockout led to death of *Lpl* deficient mice soon after birth. This was overcome through transplantation of fetal liver (Babaev et al., 1999) or bone marrow hematopoietic progenitors (Van Eck et al., 2000), as well as conditional knockout models. Eventually, multiple studies showed that *Lpl* deficiency reduces lesion size by 30%–50% in comparison to control mice. Further experiments revealed that *Lpl* expression in macrophages promotes atherogenesis, as its deficiency specifically in macrophages inhibited plaque progression (Babaev et al., 1999; Babaev et al., 2000; Takahashi et al., 2013). However, *Lpl* macrophage-specific (Chang et al., 2019) or global (Josefs et al., 2021) deficiency in established plaques did not influence atherosclerosis regression.

In the AT, *Lpl* is one of the marker genes of LAMs, together with *Trem2*, a marker for foamy (lipid-laden) macrophages in multiple tissues (Jaitin et al., 2019; Zernecke et al., 2020). TREM2 was shown to activate the lipid associated macrophages program, acting as a sensor of extracellular lipids. Early studies demonstrated that TREM2 is expressed on infiltrating macrophages but not on myeloid progenitors, circulating monocytes or tissue-resident macrophages and TREM2 suppresses proinflammatory cytokine production (Turnbull et al., 2006). *Trem2* ^*−/−*^ macrophages were recently shown to be impaired in their lipid uptake and, thus, ability to become foam cells. *Trem2* macrophage deficiency promotes their death and reduces proliferative capacity in plaques. Mechanistically, *Trem2* ^*−/−*^ macrophages have dysregulated cholesterol sensing and efflux mechanism making them susceptible to lipotoxicity-mediated cell death (Patterson et al., 2022). A study comparing serum TREM2 levels in coronary artery disease patients with healthy controls suggests that higher soluble TREM2 levels can be used as a biomarker for plaque rupture and predictor of cardiovascular death (Cuciuc et al., 2022).

### Cytokines

Several inflammatory cytokines blocking agents are currently on trial as therapy for atherosclerosis (Ridker et al., 2018; Nidorf et al., 2019; Ridker et al., 2019). The CANTOS clinical trial, featuring a monoclonal antibody against IL-1β to reduce inflammation in high-risk atherosclerosis patients did show a ∼15% decrease in recurring cardiovascular events (Ridker et al., 2017). Generally, pro-inflammatory cytokines increase cholesterol uptake, reduce efflux, promote apoptosis and impair efferocytosis. On the opposite end of the spectrum, anti-inflammatory cytokines act by reducing the cholesterol uptake and/or increasing its efflux, promoting the expression of pro-resolving proteins and mediators and inducing efferocytosis.

Anti-inflammatory cytokines can reduce cholesterol uptake in several ways. First, they inhibit pro-inflammatory cytokines, such as TNFα and IL-1β. These pro-inflammatory agents can stimulate the expression of scavenger receptors on macrophages (Hashizume and Mihara, 2012), facilitating the uptake of cholesterol and promoting foam cell formation. Anti-inflammatory cytokines, such as transforming growth factor (TGF) β, can inhibit the expression and secretion of pro-inflammatory cytokines, leading to a reduction in the expression of scavenger receptors and the uptake of cholesterol (Argmann et al., 2001; Michael et al., 2012). Second, anti-inflammatory cytokines prevent the recruitment of inflammatory cells to the arterial wall. For example, TGF-β has been shown to reduce the expression of E-selectin in endothelial cells (DiChiara et al., 2000) and VCAM1 in SMCs (Gamble et al., 1995), which are known to help immune cells bind to the endothelium and penetrate the sub-endothelial space. IL-10 also exerts its effects through inhibiting leukocyte-endothelium interaction, thus hindering their recruitment to the tissue (Downing et al., 1998; Henke et al., 2000).

Additionally, IL-10 is expressed in both early and advanced human plaques and inhibits plaque progression and rupture, NF-κB activation, metalloproteinase production and cell death (Lacraz et al., 1995; Wang et al., 1995; Lentsch et al., 1997; Mallat et al., 1999a; Mallat et al., 1999b). IL-10 acts to dampen the pro-inflammatory mediators at both transcriptional and posttranslational levels. Transgenic mice over-expressing IL-10 show reduced atherosclerotic plaques, while IL-10 deficient mice have significantly worse atherosclerosis compared to WT controls (Caligiuri et al., 2003). IL-10 was further shown to induce rearrangement of the cytoskeleton and promote apoptotic cell engulfment/efferocytosis (Proto et al., 2018). A very recent study revealed the role of macrophage derived IL-10 in limiting atherosclerosis. The authors used a combination of single-cell RNA-sequencing and flow cytometry to identify inflammatory and resident plaque macrophages as the primary source of IL-10. Myeloid specific deletion of IL-10 results in significantly larger atherosclerotic plaques along with elevated serum CCL2 and TNFα (Orecchioni et al., 2022).

IL-33 is another cytokine that seems to have a protective effect in atherosclerosis. Its levels are increased in plaques, but it is also present in normal aorta (Miller et al., 2008). IL-33 treatment was shown to reduce foam cell formation *in vivo,* while *in vitro* it reduced acetylated and oxidized LDL uptake by downregulation of *Cd36* and enhanced cholesterol efflux by upregulation of *Apoe*, *Abca1* and *Abcg1* among others. IL-33 promotes the expression of other anti-inflammatory cytokines, such as IL-4 and IL-13 (McLaren et al., 2010).

The Th2 cytokines IL-4 and IL-13 promote an anti-inflammatory macrophage phenotype through the transcription factor STAT6, and were, thus, proposed as therapies for atherosclerosis. However, similarly to other cytokines, their role is complex in disease progression and resolution. Indeed, some studies reported halted plaque progression in *Il4* single (King et al., 2002) and *Il4/Il13* double knockouts (Weinstock et al., 2021), compared to sufficient mice. In contrast, Binder and colleagues (Cardilo-Reis et al., 2012) demonstrated that *Il13*-deficiency promotes atherosclerosis and that pharmacological treatment with IL-13 induces pro-resolving macrophage polarization in plaques. These results suggest that IL-4 contributes to plaque growth, while IL-13 inhibits atheroprogression. While IL-4 seems detrimental for atherosclerosis progression, it was recently shown to be required for disease resolution (Weinstock et al., 2021). In this study we demonstrated that IL-4, but not IL-13, accumulates in plaques during disease progression, but in levels that are not sufficient to promote macrophage pro-resolving polarization. With lipid lowering, there was upregulation of the Wnt signaling pathway and PGE_2_ production, which prime macrophages to low, plaque relevant levels of IL-4. Together, Wnt and IL-4 induce macrophage pro-resolving polarization and atherosclerosis resolution (Weinstock et al., 2021).

### Lipid mediators

Low levels of SPMs were reported in plasma of patients with chronic inflammatory diseases compared to healthy controls [reviewed in (Fredman and Tabas, 2017)]. Several SPMs have been directly implicated in improving atherosclerotic plaque inflammation (Gerlach et al., 2020). Along these lines, increased SPMs in human monocyte-derived macrophages by omega-3 supplements were linked with an upregulation of macrophage phagocytosis and a decreased uptake of oxidized LDL (Sobrino et al., 2020).

COX-1, through its products, such as thromboxane A2, has been found to promote atherogenesis and deletion of *Ptgs1* (the gene encoding for COX-1) in atherogenic (*Apoe* ^*−/−*^) mice attenuates lesion development (Pratico et al., 2001). While a dual inhibition of COX-1 and COX-2 in *Ldlr* ^*−/−*^ mice reduces lesion development as well, there is still uncertainty of the involvement of COX-2 in atherosclerosis (Pratico et al., 2001). Some studies show that pharmacological inhibition and hematopoietic genetic deletion of COX-2 either inhibit (Burleigh et al., 2002), does not affect (Olesen et al., 2002; Bea et al., 2003) or promotes (Rott et al., 2003) early atherogenesis (Burleigh et al., 2002). The discrepancy might be due to the inhibitor’s mechanism of action, specificity, and concentration, as well as the target cells. Direct examination with cell type-specific knockouts will help elucidate this issue.

Major products of COX-2 are prostaglandins. PGE_2_ acts through binding to its receptors (EP1-EP4) and can direct inflammation to either strengthen or resolve it. Its functions are dependent on its concentration, target cells and additional cues available in the microenvironment (Takayama et al., 2002; Takayama et al., 2006). Lack of EP4, but not EP2 in *Ldlr* ^*−/−*^ mice fed a Western Diet for 8 weeks results in reduction in plaque area and increases apoptosis in lesions (Babaev et al., 2008), indicating processes that both harm and protect from the damages of atherosclerosis. Contrarily another study found that EP4 deficiency does not influence early atherosclerosis. At more advanced stages (after 10 weeks of Western Diet), although EP4 deficiency failed to change lesion size, it did induce pro-inflammatory gene expression (Tang et al., 2011). On the other hand, others have proposed that PGE_2_ may cooperate with IL-4 in macrophages to stimulate resolution of atherosclerosis (Weinstock et al., 2021).

SPMs were also shown to improve atherosclerosis. MaR1 and RvD2 were found to inhibit the progression of atherosclerosis by halting the increase in macrophage and necrotic core content in *Apoe* ^*−/−*^ mice (Viola et al., 2016). Others have found a marked difference in SPMs levels in vulnerable plaques compared with stable ones, including 5-LOX-derived SPMs. Specifically, there is a RvD1 deficiency in unstable plaques, coupled with increase in leukotriene B4 (LTB4). Restoration of the RvD1:LTB4 ratio in mice, by administration of RvD1, protects against advanced plaque progression (Fredman et al., 2016). This is in line with the mouse and clinical observations that plaque inflammation and vulnerability is correlated with lower levels of SPMs and a higher activity of 5-LOX, which drives the production of pro-inflammatory lipid mediators (leukotrienes) (Mehrabian et al., 2002; Spanbroek et al., 2003; Qiu et al., 2006).

### MicroRNAs

Early studies of miRNA involvement in atherosclerosis focused on their role in regulating macrophage cholesterol efflux *via* ABCA1 [reviewed in (Feinberg and Moore, 2016)]. Several were found to be significantly associated with reducing cholesterol efflux to APOA1, while their inhibition leads to increased plasma levels of HDL-cholesterol, including miR-26 (Sun et al., 2012), miR-33 (Gerin et al., 2010; Horie et al., 2010; Najafi-Shoushtari et al., 2010; Rayner et al., 2010; Rayner et al., 2011a; Rayner et al., 2011b), miR-106 (Kim et al., 2012), miR-128–1 (Wagschal et al., 2015), miR-144 (de Aguiar Vallim et al., 2013), miR-148a (Wagschal et al., 2015) and miR-758 (Ramirez et al., 2011). Among these, miR-33 is the most extensively studied, and showed promising data of atherosclerosis regression in pre-clinical models (Rayner et al., 2011b; Distel et al., 2014; Afonso et al., 2021). While there are conflicting reports about plasma HDL cholesterol levels in response to miR-33 downregulation, it was shown that the reduction in plaque size can be attributable to the pro-reparative phenotype of macrophages. Targeted deletion of miR-33 in macrophages results in enhanced oxidative phosphorylation, and anti-miR-33 treatment increases the expression of *Abca1*, *Ncoa1*, *Ncoa2*, *Crot* among other genes, all of which are involved in lipid metabolism (Afonso et al., 2021). Moreover, anti miR-33 increases macrophage’s efferocytosis capabilities (Ouimet et al., 2017).

miR-144 is also implicated in regulating lipid handling, by reducing ABCA1 levels and activity, as well as plasma HDL-cholesterol. Multiple studies have implicated miR-144 in reducing reverse cholesterol efflux in macrophages *in vitro* (Hu et al., 2014). The *in vivo* silencing of miR-144 reduces atherosclerotic lesion area in male *Ldlr* ^*−/−*^ mice but not in females. Moreover, plaque macrophages from anti-miR-144 treated mice had enhanced efferocytosis capacity *in situ*, and expressed more pro-resolving genes, such as Arginase (Arg) 1. miR-144 inhibition is associated with increased plasma HDL levels and cytochrome P450 enzyme 7B1 (oxysterol metabolizing enzyme) in livers of male mice. These results suggest a sex-specific regulation of atherosclerosis development by miR-144 (de Aguiar Vallim et al., 2013; Ramirez et al., 2013; Hu et al., 2014; Cheng et al., 2020).

Additionally, the balance between miR-342-5p and miR-155 was linked to atherosclerosis formation and pro-inflammatory phenotype of macrophages. Inhibition of miR-342-5p in cultured macrophages and *in vivo* decreased the expression of pro-inflammatory signals, such as *Nos2*, *Il6* and nitrite. Anti-miR-342-5p in a model of atherosclerosis attenuated disease progression. Mechanistically, miR-342-5p downregulates *Akt1*, which has been shown to suppress miR-155, in turn promoting inflammatory gene expression (Wei et al., 2013).

Furthermore, miR-19a-3p (Chai et al., 2020; Zhu et al., 2020), miR-124 (Liang et al., 2020), miR-146a (Nguyen et al., 2018) and miR-214 (Lu et al., 2013) have been reported to be involved in regulating macrophage inflammatory response and migration capabilities in other inflammatory diseases. However, their role in plaque macrophages remains to be elucidated.

### Efferocytosis and phagocytosis

It is thought that during the early stages of atherosclerosis, macrophages find and clear cells undergoing apoptosis in the arterial wall. As lesions progress, many vascular and immune cells die in the plaque in multiple ways such as apoptosis, necrosis and necroptosis, leading to a highly pro-inflammatory environment. The accumulation of dying cells in the plaque leads to the formation of necrotic core regions, which are thought to be the most dangerous consequence of atherosclerosis leading to plaque rupture [reviewed in (Thorp and Tabas, 2009)]. Therefore, one primary objective in atherosclerosis research is understanding the underlying mechanisms leading to defective efferocytosis in the aorta and finding therapeutic targets to improve it.

CD47 is a classical self-identification marker that prevents cells from being internalized by phagocytes and hence, considered a “don’t eat me” signal (Oldenborg et al., 2000; Gardai et al., 2005). The aberrant levels of surface CD47 in plaque apoptotic cells protects them from being cleared (i.e., efferocytosed) and results in expansion of the necrotic core (Schrijvers et al., 2005; Kojima et al., 2016). An anti-CD47 antibody has been found to reverse this defect in multiple mouse models of atherosclerosis (Kojima et al., 2016; Gerlach et al., 2020; Mueller et al., 2022). In one study, the authors found TNFα to be the main driver of increased *Cd47* expression in atherosclerotic plaques (Kojima et al., 2016). Further studies revealed that necroptotic cells have high levels of CD47, impairing their clearance in plaques by macrophages. Instead of full engulfment, necroptotic cells induce nibbling by macrophages, presumably resulting in their longer retention in the tissue. Blocking CD47 increases the production of SPMs, including RvD1, which promote the release of calreticulin from macrophages within atherosclerotic lesions (Gerlach et al., 2020). Calreticulin is a classical phagocytosis identification signal that marks cells for clearance and its upregulation is directly linked with increased efferocytosis (Gardai et al., 2005).

MerTK is important in macrophages to recognize apoptotic cells. Kinase defective MerTK in *Apoe* ^*−/−*^ mice impairs efferocytosis and increases apoptotic cell accumulation in plaques, which leads to bigger necrotic cores in advanced plaques (Thorp et al., 2008). Moreover, advanced atherosclerotic plaques have more cleavage of MerTK, resulting in impaired efferocytosis and increasing necrotic cores (Cai et al., 2017). Another important function of MerTK is the stimulation of SPM production by preventing the nuclear localization of 5-LOX (Cai et al., 2018).

Efferocytosis is not a single event process, and each macrophage needs to eat many dying cells to be able to resolve inflammation and restore tissue homeostasis. *Arg1* is a marker gene for pro-reparative macrophages and plays a key role in the metabolism of apoptotic cells. In an elegant study, Yurdagul et al. (2020) showed that ARG1 promotes the accumulation of putrescine, which enables rearrangement of the cytoskeleton to uptake more apoptotic cells. They reported that deficiency in ARG1-derived metabolites impair the ability of macrophages to efferocytose multiple cells, which can be reversed through addition of putrescine. Arginine is not the only amino acid that promotes efferocytosis. For instance, methionine was also implicated in this process, inducing the activation of PGE_2_ and subsequently TGF-β1, stimulating resolution of atherosclerosis (Ampomah et al., 2022).

## Non-alcoholic fatty liver disease (NAFLD) and non-alcoholic steatohepatitis (NASH)

NAFLD is an emerging healthcare problem worldwide and is the most common cause of chronic liver disease, affecting approximately a third of the US adult population. NAFLD can ultimately proceed to non-alcoholic steatohepatitis (NASH), characterized by liver fibrosis, and may result in cirrhosis, as well as hepatocellular carcinoma. NAFLD is a by-product of obesity and the metabolic syndrome, and even 5% loss in body weight can stimulate disease resolution (Fabbrini et al., 2010; Hannah and Harrison, 2016).

In human NAFLD, accumulation of macrophages next to the portal vein is followed by inflammatory cytokine production in the liver (Gadd et al., 2014). A causal connection of macrophages to liver pathology came from studies where clodronate liposomes or gadolinium chloride-mediated depletion of liver macrophages (Kupffer cells; KCs) led to protection from liver pathology development (Neyrinck et al., 2009; Huang et al., 2010; Stienstra et al., 2010; Chen et al., 2012). This was associated with reduction in many inflammatory parameters, including inflammatory cytokines (Stienstra et al., 2010; Tosello-Trampont et al., 2012), oxidative stress (Bleriot et al., 2021), liver cell death and hepatocyte insulin resistance (Table 3) (Huang et al., 2010; Lanthier et al., 2010).

**TABLE 3:** Summary of factors in macrophages that protect from (green) or promote (red) NAFLD/NASH.

	Factor	Function	References
**Lipid handling**	NLRP3	Enhances formation of hepatic cholesterol crystals	Hellmann et al. (2011), Pal et al. (2020)
	MSR1, CD36	Enhance hepatic inflammation, fibrosis and lipid oxidation	Bieghs et al. (2012), Govaere et al. (2022)
	PPARγ	Increases fatty acid influx to promote anti-inflammatory macrophage phenotype	Bouhlel et al. (2007), Odegaard et al. (2007), Skat-Rordam et al. (2019)
	TREM2	Prevents TLR4 mediated inflammation	Hou et al. (2021), Hendrikx et al. (2022)
**Cytokine**	TNFα, TGFβ, IL1β	Activate hepatic stellate cells thereby promoting liver steatosis, fibrosis and inflammation	Karlmark et al. (2009), Chu et al. (2013), Wree et al. (2014)
	LXR	acts as a glucose sensor and regulates insulin sensitivity and glucose homeostasis	Hirsova et al. (2016), Ibrahim et al. (2016), Tomita et al. (2016)
	FXR	Inhibits hepatic lipogenesis; enhances fatty acid oxidation and glucose and insulin sensitivity; increases production of IL-10	Claudel et al. (2005), Cipriani et al. (2010), McMahan et al. (2013), Yao et al. (2014), Verbeke et al. (2016)
	CXCL10	Induces macrophage recruitment and activation of inflammatory phenotype	Hirsova et al. (2016), Ibrahim et al. (2016), Tomita et al. (2016)
**Lipid mediators**	EPA, DHA, protectin D1, RvE1	Increase adiponectin level and insulin sensitivity	Gonzalez-Periz et al. (2009), Depner et al. (2013)
	MaR1	Reduces hepatic triglycerides and increases fatty acid oxidation which enhances autophagy	Hellmann et al. (2011), Pal et al. (2020)
**Efferocytosis/phagocytosis**	MerTK	Upregulates TGF-β1 and liver fibrosis	Hellmann et al. (2011), Pal et al. (2020)
	ADAM17	Reduces MerTK activity by its cleavage	Hellmann et al. (2011), Pal et al. (2020)
	SIRPα	Impairs clearance of necroptotic hepatocytes	Hellmann et al. (2011), Pal et al. (2020)

### Macrophage origin

KCs are mostly derived from yolk-sac-originated erythromyeloid progenitors (Schulz et al., 2012; Yona et al., 2013; Gomez Perdiguero et al., 2015; Bleriot et al., 2021) and their markers include C-type lectin domain family 4 member F (CLEC4F) (Scott et al., 2016) and T cell immunoglobulin and mucin domain containing 4 (TIMD4) (Ni et al., 2021). KCs typically self-sustain *via* local proliferation (Gomez Perdiguero et al., 2015). However, recent reports where KCs were depleted, found that monocytes can differentiate into *bona fide* KCs and take over their role (Scott et al., 2016; Remmerie et al., 2020; Seidman et al., 2020; Tran et al., 2020). Recent reports revealed that NAFLD progression is associated with the reduction in the number of embryonic-derived KCs, while monocyte-derived KCs fill in their space and function (Reid et al., 2016; Daemen et al., 2021). Interestingly, embryonic KCs die during methionine/choline deficient (MCD)-diet feeding (which promote NAFLD/NASH) and are being replaced by monocyte-derived KCs, a phenomenon that is abrogated when embryonic KCs are protected from death. Monocyte-derived KCs were further shown to assume the phenotype of embryonic-derived KCs following the discontinuation of the MCD diet (Tran et al., 2020).

The newly recruited, monocyte-derived KCs are generally more pro-inflammatory, thus promoting liver damage, while also being less efficient in triglyceride storage (Tran et al., 2020). Apart from replacing embryonically originated KCs, recruited monocytes differentiate into macrophages specialized in lipid handling that resemble the LAMs in adipose (Remmerie et al., 2020). These hepatic LAMs were identified as the ones forming crown-like structures in the liver and their loss caused hepatic fibrosis (Daemen et al., 2021). These two monocyte-derived KC types have different capacity for lipid handling possibly because they are distinct subpopulations, each specializing in discrete functions. While monocytes downregulate *Ccr2* and increase *Clec4a* expression upon acquisition of a KC phenotype, they never upregulate *Timd4* expression (Sakai et al., 2019; Remmerie et al., 2020), indicating that *Timd4* might be a good marker to discern KC origin.

Recruitment of monocytes to the liver in the context of NAFLD is primarily through CCR2. The expression of CCR2 in livers is significantly correlated with body weight in ob/ob and DIO mouse models (Obstfeld et al., 2010; Parker et al., 2018). *Ccr2* expression was found to be 5-fold higher in recruited monocytes compared to resident KCs, while its ligand, *Ccl2*, was increased in KCs compared to monocyte-derived liver macrophages (Morinaga et al., 2015). NAFLD is associated with a significant increase in recruited monocytes that assume a pro-inflammatory phenotype in comparison to control mice (Morinaga et al., 2015). The high expression of *Ccl2* by KCs indicates that they promote the recruitment of monocytes to the liver in obese/NAFLD conditions. Genetic deletion and pharmacologic inhibition of CCR2 in mouse models of NAFLD/NASH led to recruitment of fewer monocytes and improved liver pathology (Weisberg et al., 2006; Yang et al., 2009; Baeck et al., 2012; Miura et al., 2012). Additionally, depletion of KCs decreases the infiltration of monocytes to the liver, which was seen as early as 6 days after initiation of a NASH-inducing MCD diet, possibly due to the reduction in CCL2 and TNFα (Tosello-Trampont et al., 2012). Moreover, RNA-sequencing of hepatic macrophages from MCD diet-fed mice revealed upregulation of inflammatory cytokines in embryonic and recruited macrophages, with monocyte-derived KCs being more enriched in growth factors for angiogenesis and liver fibrosis, as well as genes involved in phagocytosis (Krenkel et al., 2018). Hence, KCs are essential for the initial NAFLD phases and are the primary source of inflammatory cytokines and chemoattractants responsible for the recruitment of pro-inflammatory monocytes to further disease progression. However, given our knowledge of important lipid handling and inflammation resolution properties of monocyte-derived macrophages in other tissues, we postulate there are similar beneficial macrophage populations in the liver yet to be fully appreciated.

### Lipid handling

Obesity promotes the activation of macrophages in the liver and other tissues *via* multiple triggers, including endotoxins, cholesterol and its metabolites, fatty acids, and danger-associated molecular patterns from hepatic damage. Since the liver is responsible for packaging dietary and *de novo* synthesized lipids in cholesterol-rich particles, high fatty acid surplus (due to obesity, for instance), causes lipotoxic stress in all cell types of the liver. Lipotoxicity in hepatocytes promotes their secretion of extracellular vesicles that contain inflammatory stimuli. These include CXCL10, which induce macrophage recruitment and inflammatory activation (Hirsova et al., 2016; Ibrahim et al., 2016; Tomita et al., 2016). Additionally, dietary peroxidised lipids possibly induce a pro-inflammatory environment in the liver. This was demonstrated *via* feeding peroxidised corn oil to rats, which increases hepatic lipid oxidation. *In vitro*, peroxidised linoleic acid (main fatty acid in corn oil) treated KCs upregulate pro-inflammatory gene expression, such as *Nos2*, *Ptgs2* (gene name of COX-2) and *Tnfα*, through p38 kinase activation (Bohm et al., 2013).

In hyperlipidemic conditions, KCs become foamy, similarly to foam cells in atherosclerotic plaques and adipose, due to their accumulation of free cholesterol, as well as uptake of lipids from dead hepatocytes. KCs form crown-like structures in the liver, which were proposed to distinguish NAFLD from NASH in humans (Ioannou et al., 2013). WT mice fed a moderate-fat (15%), high cholesterol (≥0.5%) diet for 6 months developed hepatic cholesterol crystals which were surrounded by crown-like structures, consisting of KCs which were NLRP3 and caspase-1 positive. Additionally, HepG2 cells (hepatocyte cell line) treated with LDL and oleic acid develop cholesterol crystals, which upon co-culturing with THP-1 cells (monocyte cell line) lead to increased *Tnfa*, *Nlrp3* and *Il1b* expression in the hepatocytes. The authors suggest that NLRP3 activation is the link between cholesterol and chronic inflammation, which leads to the development of NASH (Ioannou et al., 2017). Although promising as a possible therapy, NLRP3 gene deletion or pharmacological inhibition was recently reported to have no influence on NASH (Ioannou et al., 2023).

Uptake and clearance of lipoproteins from the circulation *via* macrophage scavenger receptor 1 (MSR1) and CD36, while important, was shown to be deleterious for the NAFLD pathology. Hematopoietic deletion of both these genes in *Ldlr* ^*−/−*^ mice resulted in reduced hepatic inflammation, fibrosis and lipid oxidation, with no difference in the number of foamy KCs (Bieghs et al., 2010). Similar results were also obtained with single knockouts for *Msr1* and *Cd36* (Bieghs et al., 2012). The roles of these scavenger receptors lie not only in lipid uptake, since the reduction in inflammation with the deficiency of both genes did not correspond to a reduction in cellular lipid (Bieghs et al., 2012). That said, they contribute independently to the initiation of inflammation *via* modulating KC intracellular cholesterol distribution, plausibly impairing cellular functions (Bieghs et al., 2012). *Msr1* transcript levels were found to correlate with the degree of steatosis and steatohepatitis in human NAFLD patients and a polymorphism upstream of *Msr1* was strongly correlated with aspartate aminotransferase and serum triglyceride levels (Govaere et al., 2022). Taking all these data together, it seems that MSR1 and CD36 regulate the quality and distribution, and not quantity, of lipids in liver macrophages. Further understanding of these pathways may assist in finding more efficient treatments for NAFLD/NASH.

PPARγ also plays a crucial role in diverting macrophages to an anti-inflammatory phenotype by increasing fatty acid influx and oxidation [reviewed in (Skat-Rordam et al., 2019)]. Dysfunctional AT promotes lipolysis and hyperlipidemia in response to HFD, which makes the liver act as a secondary reserve for the excess lipid load. This results in the increased expression of *Pparg* in the liver, and subsequent hepatic triglycerides accumulation. Moreover, liver specific *Pparg* knockout in ob/ob mice causes ∼60% increase in serum free fatty acids, presumably because the liver can no longer store as many lipids, promoting insulin resistance and ectopic fat accumulation in the muscle (Matsusue et al., 2003). PPARγ activation plays an important role in macrophage polarization and is critical to switch macrophages to anti-inflammatory phenotype (Bouhlel et al., 2007; Odegaard et al., 2007). Rosiglitazone, a synthetic ligand of PPARγ, has been trialed to treat NASH with disappointing results (Ratziu et al., 2010), yet was found to skew KC polarization towards a resolving phenotype *in vitro* and in mice (Luo et al., 2017). A myeloid *Pparg* deletion (on a Balb/c background) exacerbates insulin resistance in liver and muscle, without obvious signs of steatohepatitis (Odegaard et al., 2007). In another mouse model of NAFLD, myeloid-specific *Pparg* deletion caused an increase in pro-inflammatory cytokines and worsened fibrosis in comparison to WT controls (Moran-Salvador et al., 2013). Together these data suggest that PPARγ activation in macrophages might be considered as a therapeutical target, however, it is important to ensure sufficient availability to macrophages.

There is some evidence to suggest that TREM2 may be involved in the development of NAFLD. Like in atherosclerosis, the systemic levels of soluble TREM2 are found to be elevated in humans and mice with NAFLD. TREM2 expressing KCs localize to sites of hepatic damage, inflammation and fibrosis, and soluble TREM2 correlates with disease severity in humans. Additionally, *Trem2* deficiency exacerbates liver inflammation and fibrosis, indicating that it functions to mitigate NASH-related pathologies (Hendrikx et al., 2022). One study uncovered that *Trem2* deficient macrophages impair hepatic mitochondrial structure and energy supply, thus accelerating NAFLD initiation (Hou et al., 2021). All the above studies suggest that *Trem2* macrophage expression is beneficial in the liver, and may prevent hepatic damage, similarly to its potential role in AT.

### Cytokines

TNFα (De Taeye et al., 2007) and IL-1β (*via* NLRP3 and caspase-1) (Wree et al., 2014) drive liver steatosis, inflammation and fibrosis. TNFα and TGFβ activate hepatic stellate cells, thus promoting liver fibrosis (Karlmark et al., 2009; Chu et al., 2013). Immune cells produce these inflammatory cytokines in the liver through the transcription factor c-Jun N-terminal Kinase-1 (JNK1). Deficiency in *Jnk1* (but not *Jnk2*) in hematopoietic cells protects from liver fibrosis and inflammation and improves insulin resistance, without influencing lipid accumulation (Solinas et al., 2007; Kodama et al., 2009). NAFLD/NASH also results in increased inflammatory markers in the plasma (e.g., IL-6 (Wieckowska et al., 2008), IL-8 (du Plessis et al., 2015; Jamali et al., 2016), TNFα, ICAM-1 and VCAM-1 (Wieckowska et al., 2008; du Plessis et al., 2015; Jamali et al., 2016; Sookoian et al., 2010). Soluble CD163 (which is mainly produced by macrophages) predicts liver fibrosis, and its level correlates with severity of fatty liver disease in humans (Kazankov et al., 2015; Kazankov et al., 2016).

AT can promote NAFLD development *via* cytokine and adipokine secretion, which affects systemic metabolism [reviewed in (Schaffler et al., 2005)]. Even more so, some studies suggest that AT dysfunction serve as a precursor to insulin resistance and NAFLD (Bugianesi et al., 2005; Stanton et al., 2011). Furthermore, inflammatory macrophages were shown to impair hepatocyte responsiveness to insulin, thus promoting hepatic and systemic insulin resistance (Huang et al., 2010). Interestingly, a recent study suggests that ATMs induce hepatic macrophage accumulation. The authors transplanted visceral AT from lean, obese, or ATM-depleted obese mice into lean *Ldlr* ^*−/−*^ recipients. After 8 weeks on high cholesterol diet, there was an increase in hepatic macrophages and neutrophils in the obese AT recipient, in comparison to lean or ATM-depleted AT recipients (Bijnen et al., 2018), showing direct contribution of ATMs to accumulation of immune cells in the liver. However, there were no apparent differences in liver pathology, possibly due to the short-term feeding of high-cholesterol diet.

LXRs are transcription factors that belong to the nuclear hormone receptor family. They play a role in the regulation of lipid metabolism and inflammation in the liver [reviewed in (Hong and Tontonoz, 2014)]. It acts as a glucose sensor and regulates insulin sensitivity and glucose homeostasis (Mitro et al., 2007). Although LXRs have been shown to increase in livers of NAFLD patients (Higuchi et al., 2008), they are believed to have a protective effect against disease worsening. In animal models of NAFLD, activation of LXRs has been shown to reduce the accumulation of fat in the liver and improve insulin sensitivity (Beaven et al., 2013; Griffett et al., 2013). In addition, LXRs have been shown to have anti-inflammatory effects in the liver, which may also contribute to their protective effects against NAFLD (Hamilton et al., 2016). LXRs can also reduce the proinflammatory effects of TNFα and iNOS through inhibiting Phosphoinositide 3-kinase and JNK signaling to attenuate LPS-mediated liver injury in mice with NAFLD (Liu et al., 2012). Conversely, LXR systemic activation have been associated with hypertriglyceridemia and hepatic steatosis [reviewed in (Fessler, 2018)]. This could be due to different effects of the LXR isoforms, which require further investigation.

Farnesoid X receptor (FXR) is a nuclear receptor activated by bile acids, which promote its transcription factor activity. FXR inhibits hepatic lipogenesis, instead inducing fatty acid oxidation [reviewed in (Claudel et al., 2005)]. In rodents, FXR engagement was shown to inhibit NAFLD-related liver pathologies, as well as protect from insulin and glucose intolerance (Cipriani et al., 2010; Verbeke et al., 2016). FXR agonism in diabetic obese mice reduces liver expression of genes related to inflammation and fibrosis by promoting the accumulation of Ly6c^lo^ monocytes and increases production of IL-10 (Cipriani et al., 2010; McMahan et al., 2013; Yao et al., 2014; Verbeke et al., 2016). Benefits of FXR activation on liver pathology and insulin sensitivity were also found in randomized, placebo-controlled clinical trials (Mudaliar et al., 2013; Neuschwander-Tetri et al., 2015), which are partly due to decreased hepatic lipid absorption (Clifford et al., 2021). That said, a recent study showed that these benefits of FXR are agonist dose dependent, as high doses in mice promoted liver fibrosis.

### Lipid mediators

The abundances of hepatic lipid mediators are altered in NASH patients, with specific decrease in arachidonic acid, as well as EPA and DHA (Puri et al., 2007). Even more so, feeding EPA and DHA enriched diet prevents the development of severe hepatic steatosis and increases adiponectin levels and insulin sensitivity in obese mice. These beneficial effects were replicated by treatment with RvE1 and protectin D1 (Gonzalez-Periz et al., 2009). Another study reported that DHA reduces hepatic inflammation, oxidative stress and fibrosis in hypercholesterolemic *Ldlr* ^*−/−*^ mice but did not reduce liver fat accumulation. The authors compared the effects of dietary DHA and EPA and find that DHA more profoundly attenuates Western Diet-induced liver expression of genes related to inflammation and fibrosis (Depner et al., 2013).

One study examined the effects of combining weight loss with RvD1 administration on NASH. The authors found that switching DIO mice to normal chow increases adiponectin levels and reduces macrophage numbers in the liver. RvD1 also attenuates the pro-inflammatory signature and induces pro-reparative macrophage phenotype, beyond the levels of weight loss alone. The benefits of RvD1 were lost following macrophage depletion, indicating that RvD1 exerts its effect through macrophage modulation (Rius et al., 2014).

NAFLD was also associated with decreased serum MaR1 levels in humans (Fang et al., 2021). Treatment with MaR1 reduces hepatic triglycerides, while increasing fatty acid oxidation gene expression and autophagy in ob/ob and DIO mice (Laiglesia et al., 2018). Additionally, MaR1 prevents ER stress in palmitate-induced lipotoxicity of hepatocytes and blocks the upregulation of proapoptotic pathways (Rius et al., 2017). MaR1 acts as an endogenous ligand for Retinoic acid–related orphan receptor α (RORα), a key regulator of macrophage pro-resolving polarity. RORα activation by MaR1 promotes the transcription of 12-LOX, resulting in the production of more MaR1, thus establishing a feed-forward loop of MaR1 synthesis. This resulted in protection of mice from high-fat diet induced NASH in a RORα dependent manner. Finally, it was found that liver macrophages from NASH patients had lower expression of 12-LOX than those from healthy controls (Han et al., 2019).

Although the data above indicate that SPMs might be beneficial in treating NAFLD/NASH, evidence in humans is disappointing so far. An early small pilot study and phase I clinical trial treated patients with omega-3 PUFA (Capanni et al., 2006) or highly purified EPA (Tanaka et al., 2008), respectively, showed decreased liver fat compared to baseline levels. However, several double-blinded, placebo-controlled trials showed marginal improvement to no change in liver fat content, with no change in liver fibrosis (Sanyal et al., 2014; Scorletti et al., 2014; Nogueira et al., 2016). These results indicate that treatment with SPM precursors may not be beneficial to treat NAFLD/NASH. However, SPMs themselves might still prove efficient as therapies in the future.

### MicroRNAs

There have been multiple studies that identified and quantified circulating miRNAs in human NAFLD patients and linked them to disease severity. One study evaluated the expression of 84 circulatory miRNAs and found that miR-122, miR-192, miR-19a/b, miR-125b and miR-375 were upregulated >2-fold in NAFLD (Pirola et al., 2015). In another study, a panel of several circulating miRNAs (e.g., miR-122-5p, miR-1290, miR-37-3p, miR-192-5p), was suggested to predict NAFLD diagnostically (Tan et al., 2014).

miR-155 is a master regulator of inflammation in multiple diseases and has increased expression in murine livers of MCD diet-induced steatohepatitis (Csak et al., 2015). miR-155 is increased in hepatocytes as well as mononuclear cells and KCs. miR-155 was thought to exert its effects through TNFα, however, miR-155 deficient mice do not show altered levels of *Tnfa*, *Ccl2* and *Il1b*, with no changes to liver inflammation, while improving liver lipid accumulation and fibrosis (Csak et al., 2015).

As discussed in previous sections, miRNAs can serve as intercellular and inter-tissue communicators when they are packaged and sent *via* exosomes. Serum miR-192-5p, derived from lipotoxic hepatocyte-secreted exosomes, was found to be positively correlated with hepatic inflammatory activity score and disease progression in human NAFLD patients. In a high-fat high-cholesterol diet-fed rat model, lipotoxic hepatocytes were shown to release more miR-192-5p enriched exosomes. This induces pro-inflammatory macrophage activation along with an increased expression of *Nos2*, *Il6* and *Tnfa*. miR-192-5p activates a pro-inflammatory program in macrophages through a signaling pathway involving Rictor, AKT, and Forkhead Box transcription factor O1 (Liu et al., 2020).

miR-690 is another NAFLD-associated exosome-derived miRNA and is believed to be protective. Levels of miR-690 are markedly lower in human and mouse NASH livers in comparison to healthy controls. KCs are the main source of miR-690 in the liver, which is delivered *via* exosomes to other liver cells such as hepatocytes, macrophages and hepatic stellate cells, where it inhibits *de novo* lipogenesis, inflammation and fibrogenesis respectively. Mechanistically, miR-690 mimic administration and upregulation *in vivo* inhibited NASH phenotype *via* targeting NADK mRNA to elevate NAD^+^ levels. Replenishment of NASH mice with NAD^+^ attenuated disease pathogenesis (Gao et al., 2022).

### Efferocytosis and phagocytosis

Liver cell death comes in many forms (i.e., apoptosis, necrosis, pyroptosis, ferroptosis and more recently necroptosis), all of which lead to inflammation and fibrogenesis [reviewed in (Malhi et al., 2010)]. Professional phagocytes, particularly macrophages, play a critical role in removing dead hepatocytes to mitigate inflammation and stimulate TGFβ release (Schwabe and Luedde, 2018). While efferocytosis is considered protective, release of TGFβ results in the activation of hepatic stellate cells and subsequently fibrosis (Kim et al., 2022).

One of the major receptors for dying cells is MerTK, which is imperative for the efferocytic process, is described above. Human genome-wide association study also implicated MerTK as an important factor in liver fibrosis (Patin et al., 2012). Surprisingly, mechanistic studies show that MerTK signaling in liver macrophages promotes NASH development *via* ERK1/2-mediated upregulation of TGF-β1. In turn, TGF-β1 activates collagen synthesis in hepatic stellate cells and promotes fibrosis. ADAM17 mediated MerTK cleavage in early, non-fibrotic steatosis is a protective process, presumably *via* decreasing MerTK’s receptor activity, which is reduced in disease progression (Petta et al., 2016). Moreover, cleavage resistant mutant MerTK enhances NASH fibrosis, while all-trans retinoic acids block MerTK activation *via* ADAM17-induced cleavage, resulting in suppression of NASH progression (Cai et al., 2020). ADAM17 was also recently shown to be produced in the liver in response to prolonged hyper-nutrition, where it cleaves TREM2. Reduction in membranal TREM2 results in accumulation of dead hepatocytes due to impaired efferocytosis by KCs (Wang et al., 2023). Together, these data suggest that efferocytosis is imperative for reduction of obesity-related liver inflammation, however, the specific mechanisms by which KCs efferocytose are a crucial determining factor of liver pathology.

Necroptosis of hepatocytes adds a layer of burden on the debris clearing macrophages. It was recently reported that necroptotic, but not apoptotic, hepatocytes upregulate the expression of the “don’t-eat-me” signal CD47. At the same time, liver macrophages increase their expression of the CD47 receptor, SIRPα, leading to impaired clearance of these necroptotic hepatocytes. This was reversed using anti-CD47 or anti-SIRPα treatment in mouse models of NASH, which increased the uptake of these necroptotic hepatocytes by liver macrophages (Shi et al., 2022).

## Neuroinflammation

It has been appreciated for a long time that obesity is a risk factor for stroke (Strazzullo et al., 2010). More recently, evidence has linked obesity with several neurological defects, such as deficits in learning and memory (Erion et al., 2014), dementia (Singh-Manoux et al., 2018) and Alzheimer’s disease (AD) (Nianogo et al., 2022). The hallmark of AD is the accumulation of amyloid beta (Aβ) plaques in the brain, leading to neurofibrillary tangles, neuronal dysfunction/loss, which eventually results in dementia and death.

Neuroinflammation is a response aimed at protecting the central nervous system (CNS). Within the brain, this response can be elicited by resident macrophages known as microglia, however, prolonged neuroinflammation is harmful and can lead to brain damage. Pronounced neuroinflammation can be observed in neurodegenerative diseases, most evidently by increased microglial activation.

Microglia are constantly monitoring the microenvironment to respond to any kind of pathological change, as well as maintaining tissue homeostasis (Li and Barres, 2018). They represent 10%–20% of glia population in the brain, similar to the number of neurons, and their markers include Iba1, TMEM119 and P2RY12 (Kenkhuis et al., 2022). In physiological conditions, microglia maintain synaptic structure, remodel presynaptic environments (Miyamoto et al., 2016; Weinhard et al., 2018; Du et al., 2022), promote neurogenesis (Thored et al., 2009), induce retinal angiogenesis (Checchin et al., 2006), and release some neurotrophic factors. In addition, microglia act as phagocytes, removing cells undergoing apoptosis (Anderson et al., 2022). Microglia have a complex response to nutritional overload, including cell activation and proliferation, which may increase or reduce inflammation in neurodegenerative diseases. Microglia responds to obesity by producing and releasing cytokines and other mediators which alter neuronal functions (Table 4).

**TABLE 4:** Summary of factors in macrophages that protect from (green) or promote (red) neuroinflammation.

	Factor	Function	References
**Lipid handling**	TREM2	Enhances myelin phagocytosis and regulates autophagy to prevent inflammation	Paloneva et al. (2002), Atagi et al. (2015), Nugent et al. (2020)
	LPL	Enhances phagocytosis and Aβ uptake; prevents weight gain and degradation of myelin	Ma et al. (2013), Cantoni et al. (2015), Leng et al. (2022)
	HK2	Negative regulator of LPL	Leng et al. (2022)
**Cytokine**	IL-1β	Promotes microglia accumulation; impairs memory	Heneka et al. (2013)
	IL-4	Inhibits the expression of pro-inflammatory cytokine and TLR4	Guo et al. (2021)
	TAK1	Promotes excess production of IL-18 and enhances cerebrovascular dysfunction	Shen et al. (2020)
	TNFα, IL-1α, Clq	Promotes neuronal death by inducing astrocytes activation	Liddelow et al. (2017)
**Lipid mediators**	PGE_2_	Prevents satiety	Niraula et al. (2022)
	MaR1	Enhances phagocytosis of Aβ	Zhu et al. (2016)
	N-AS, COX-2	Increase phagocytosis	Lee et al. (2020)
**miRNA**	miR-27a	Decreases production of pro-inflammatory cytokines by suppressing the expression of *Tlr4*	Lv et al. (2017)
	miR-155	Decreases SOCS-1 production thereby promoting inflammation	Cardoso et al. (2012), Woodbury et al. (2015)
**Efferocytosis/phagocytosis**	STAT6, ARG1, GAS6	Enhance efferocytosis, reduce production of pro-inflammatory marker and protect against neuronal death	Cai et al. (2019), Tang et al. (2022)
	LPL	Increases production of ATP to fuel phagocytosis	Bieghs et al. (2012), Govaere et al. (2022)
	TREM2	Enhances microglia proliferation and myelin debris clearance	Cantoni et al. (2015)
	HK2	Prevents phagocytosis and enhances accumulation of Aβ	Leng et al. (2022)

### Microglia origin

The origin of microglia has been of interest because of its pharmacological potential in neuroinflammatory conditions. Microglia are derived from yolk-sac primitive macrophages that seed the embryo’s brain and are maintained mostly by local proliferation (Ginhoux et al., 2010; Yona et al., 2013). Microglia proliferate more in AD brains, in both mouse models and post-mortem patient samples (Olmos-Alonso et al., 2016). Inhibition of microglia proliferation and survival using anti-colony-stimulating factor 1 receptor antibody protects from synapse degeneration and improves memory in a mouse model of AD (Olmos-Alonso et al., 2016).

Although microglia are mostly of embryonic origin in steady state, they may also be derived from monocytes in some conditions. Recent findings indicate that the skull contains myeloid progenitors that produce monocytes and neutrophils and act as a local source of these cells in the brain. These skull monocytes can undergo differentiation to form meningeal macrophages, which proliferate and move into the CNS in inflammatory conditions and following injury (Cugurra et al., 2021). Other studies also supported that bone marrow-derived cells infiltrate the CNS during HFD and become microglia. One such study demonstrates that bone marrow-derived cells move into the hypothalamus during HFD through TLR4 and fractalkine signaling and the latter is elevated in the hypothalamus of obese mice. Inhibition of hypothalamic fractalkine decreases the local expression of inflammatory cytokines induced by HFD and protects from DIO (Morari et al., 2014). Conversely, others showed that microglia numbers significantly correlate with BMI in humans and increase in HFD-fed mice through local proliferation, rather than recruitment of monocytes (Baufeld et al., 2016).

Monocytes do not only infiltrate the CNS during obesity, but also during the pathogenesis of a number of neurological disorders. In a study carried out in an ischemic stroke mouse model, blood Ly6C^hi^ monocytes were found to migrate into the brain after ischemia and differentiate into macroglia. Post-induction of ischemic stroke, the authors transferred monocytes harboring a red fluorescent protein, or a CD45.1 congenic mismatch. They observed that Ly6C^hi^ monocytes that infiltrate the brain are localized near blood vessels and assume an anti-inflammatory phenotype (Miro-Mur et al., 2016). A newer study also supported these data, using fate mapping of bone-marrow derived myeloid cells and single-cell RNA-sequencing, showing that in AD, microglia are both embryonic and monocyte-derived (Silvin et al., 2022). In contrast, it was recently reported that in AD, plaque-associated microglia are derived from resident, yolk-sac origin, with no contribution from monocytes. This was concluded after a series of experiments in monocyte (CCR2-Cre) and microglia (CX_3_CR1-Cre) fate mapping mice, crossed to an AD mouse model. It was found that >98% of microglia in AD brains came from CX_3_CR1+ microglia, and not from CCR2+ monocytes (Reed-Geaghan et al., 2020). In conclusion, there is still debate about the origin of microglia in neurological disorders. A novel mouse model may help elucidate this important issue, in which microglia can be targeted and labeled, without affecting monocytes or parenchymal macrophages (Kim et al., 2021), and it is to be seen what this interesting tool will yield.

### Lipid handling

The brain structure and function depend on *de novo* synthesis and intercellular exchange of lipids. Change in microglial lipid homeostasis is related to the development of several neurological diseases. Microglia regulate the susceptibility to diet induced obesity and link dietary overconsumption to hypothalamic dysfunction (Valdearcos et al., 2017).

In the CNS, TREM2 is mainly expressed by microglia, where it senses lipids, mediates myelin phagocytosis and regulates autophagy. The same group that first described *Trem2*-dependent LAMs in the AT also previously found a similar protective microglia population in AD brains that highly expresses *Trem2* (Keren-Shaul et al., 2017). These cells were termed disease-associated microglia (DAMs), as they were found in several human neurodegenerative diseases. Using time response assays, they further demonstrated that DAMs are first activated independently of *Trem2*. However, in the late stages of AD, *Trem2* is needed to activate protective programs of lipid metabolism and phagocytosis (Keren-Shaul et al., 2017), although others suggested that *Trem2* is required for microglia transition to DAMs (Nugent et al., 2020). Subsequent studies also showed that *Trem2* deficiency or loss of function impairs lipid metabolism leading to neuroinflammatory diseases (Paloneva et al., 2002). Additionally, TREM2 reduces pathologies of demyelination-associated neurodegenerative diseases by phagocytosing myelin. It was recently shown that phagocytosis of myelin, which contains >80% of the brain’s free cholesterol, is mediated by cholesterol uptake and metabolism in microglia, through TREM2’s activity. An important ligand of TREM2 is APOE, a major component of several types of lipoprotein particles. TREM2 variants associated with human AD are impaired in their APOE binding capacity, indicating that this interaction is crucial for neurological health (Atagi et al., 2015). Microglia from *Trem2* ^*−/−*^ mice have increased cholesteryl ester levels and fail to upregulate *Apoe*, suggesting skewed lipid uptake and handling (Nugent et al., 2020).

*Trem2* ^*−/−*^ microglia also show lower *Lpl* expression, which was proposed to lead to defective myelin degradation by microglia (Cantoni et al., 2015). *In vitro* investigations demonstrate that microglial LPL is necessary for the uptake of amyloid β (Aβ) but not its intracellular degradation (Ma et al., 2013). Furthermore, it was recently found that hexokinase (HK) 2, the rate limiting enzyme in glucose metabolism, is a negative regulator of microglial LPL. Genetic ablation of HK2 in microglia increases the level of triglycerides and LPL, which stimulates free fatty acid metabolism to produce ATP, leading to Aβ uptake and improved cognitive functions in a mouse model of AD (Leng et al., 2022). HFD itself was found to increase hypothalamic microglia *Lpl* gene expression too. *Lpl* knockout in microglia drives a metabolic shift in mitochondria, utilizing glutamine instead of lipids. Under high-fat high-carbohydrate conditions, *Lpl* deficiency enhances weight gain and glucose intolerance, reduces phagocytic activity and mitochondrial dysmorphia, indicating microglial LPL maintains hypothalamic integrity in obesity (Gao et al., 2017).

### Cytokines

HFD leads to an increase in serum levels of IL-1β and TNFα, as well as macrophage infiltration into mouse brains. A recent study investigated the contribution of beige adipocytes, which reside subcutaneously but not in visceral fat, to cytokine production and neuroinflammation. The authors used a conditional knockout in adipocytes of *Prdm16*, a master regulator of adipocyte beiging (the process in which white adipocytes acquire a metabolically active phenotype, resembling brown adipocytes). Results show that the observed increases in serum IL-1β (but not TNFα) under HFD are more pronounced in mice lacking beige fat, compared with mice having normal beige fat. Macrophage infiltration to brains in beige fat-deficient mice was noticed, and accompanied by pro-inflammatory activation of microglia, resulting in early onset hippocampal dysfunction. Intriguingly, transplantation of subcutaneous AT from lean mice to DIO WT mice restore cognitive deficits, while AT transferred from *Prdm16* ^*−/−*^ failed to show this improvement*.* Mechanistically, beige fat induced the expression of the IL-4 receptor in microglia and IL-4 secretion from brain T cells, promoting an anti-inflammatory state of microglia (Guo et al., 2021). IL-1β has also been implicated in memory deficit in obese mice. Increase in microglia number and hippocampal IL-1β was observed in db/db mice, leading to impairment in memory function, a phenotype that can be reversed by treadmill exercise. Impaired memory is also observed in lean mice that were transplanted with AT from db/db mice. The level of IL-1β in these mice correlate with adipose mass and cognition, while inhibition of hippocampal IL-1β in db/db mice restores memory function (Erion et al., 2014). Additionally, a recent study showed that IL-1β produced by NLRP3-activated visceral AT might be responsible for obesity-related cognitive impairment. Using transplantation of AT, the authors demonstrated that transfer of WT, but not *Nlrp3* ^*−/−*^ obese AT causes deficiencies in hippocampus-dependent memory and synaptic plasticity. These cognitive impairments were attributed to activation of the IL-1 receptor in microglia, since its conditional knockout alleviated obesity-induced neuroinflammation (Guo et al., 2020). Furthermore, *Nlrp3* ^*−/−*^ mice are protected from AD-associated cognitive declines (Heneka et al., 2013).

Interestingly, Baufeld et al. (2016), found that IL-1β is only upregulated in hypothalamic microglia during short-term (3 days) HFD, an increase that is not observed at 8 weeks of HFD feeding. Alternatively, chronic HFD feeding causes an upregulation in anti-inflammatory factors, such as PPARγ and IL-10. Prolonged HFD is also associated with hyperactivation of transforming growth factor β-activated kinase 1 (TAK1) in brainstem microglia, thereby promoting the overproduction of IL-18. Activation of the TAK1/IL-18 pathway results in cerebrovascular dysfunction and might be one of the underlying causes of stroke. Pharmacologic inhibition and microglia-selective genetic deletion of *Tak1* results in improved cerebrovascular function and histopathology (Shen et al., 2020).

Microglia, through release of cytokines (e.g., TNFα, IL-1α, and C1q; Complement component 1q) have been shown to induce astrocytes activation, disabling their neuroprotective functions and, thus, promoting neuronal death. LPS-stimulated microglia secrete IL-1α/β, TNFα and C1q, which leads to the activation of astrocytes. Reactive astrocytes lose normal functions, including decreased synaptic functions and phagocytic capacity. This results in death of neurons and oligodendrocytes, manifesting in neurodegeneration. This astrocyte phenotype was found in many human neurodegenerative conditions, including AD, Huntington’s, Parkinson’s and multiple sclerosis (Liddelow et al., 2017).

### Lipid mediators

PGE_2_ activation of microglia was recently shown to directly promote obesity and metabolic derangement. Conditional knockout of the PGE_2_ receptor EP4 specifically in microglia protects mice from DIO and improves insulin intolerance, probably by reducing appetite and food intake. Mechanistically, it was demonstrated that EP4 sufficient, but not deficient, microglia phagocytose Proopiomelanocortin neurons. These hypothalamic neurons are responsible for satiety, and their elimination by microglia (which is mediated by EP4) causes overeating and subsequent metabolic dysfunction (Niraula et al., 2022).

The decrease in SPM levels in AD brains negatively affects inflammation resolution. Lower levels of LXA4 were found in the cerebrospinal fluid and hippocampal tissue of AD patients. The abundance of LXA4 and RvD1 are positively correlated with cognitive function, all of which were reduced in AD patients. This was also coupled with an increase in levels of multiple PGs and a decrease in IL-10 in AD brains compared to controls (Wang et al., 2015). In a follow-up study, the same team found reduction in RvD5, MaR1 and protectin D1 as well as increased PGD_2_ in the entorhinal cortex of AD patients, compared to healthy controls. *In vitro*, MaR1 was found to increase microglia engulfment of Aβ and reduce their pro-inflammatory activation (Zhu et al., 2016).

SPMs were also shown recently to improve AD in mice. Sphingosine kinase1 uses N-acetyl sphingosine (N-AS) to acetylate COX-2. Reduction in microglial N-AS in AD leads to decreased acetylated COX-2 and subsequent SPM production (specifically 15R-LXA4, RvE1, and RvD1). Treatment of AD mice with N-AS increases COX-2 triggered SPMs in microglia, leading to inflammation resolution, increase in microglial phagocytosis and improved memory. This suggests that alteration in microglia N-AS generation and reduction in acetylated COX-2/SPM drive the defects that worsens AD (Lee et al., 2020).

### MicroRNAs

Dysregulation of miRs is suspected to contribute to microglial hyper-activation, abnormal microglia polarization and persistent neuroinflammation. An *in vitro* study found that miR-27a expression is rapidly decreased in microglia after LPS stimulation. Knockdown of miR-27a increases the expression of inflammatory cytokines (e.g., *Il6*, *Il1b* and *Tnfa*). Along these lines, overexpression of miR-27a decreases the production of inflammatory cytokines and suppresses the expression of *Tlr4* and interleukin-1 receptor-associated kinase 4 by directly binding their 3′–UTRs (Lv et al., 2017). Another *in vitro* study observed upregulation of microglial miR-155 expression coupled with a decrease in the levels of the suppressor of cytokine signaling 1 (SOCS-1), an inhibitor of LPS-induced inflammation. Knockdown of miR-155 upregulates SOCS-1 levels and leads to decreased production of nitric oxide and expression of inflammatory cytokines (e.g., *Ifnb*, *Tnfa*, and *Il6*) (Cardoso et al., 2012; Woodbury et al., 2015).

The ability of miR-424 to lessen ischemic brain injury has also been reported. miR-424 is reduced in patients and mice with ischemic stroke and this is associated with an increase in pro-inflammatory cytokines and activation of microglia, which leads to an increase in infarct volume. Cerebroventricular-overexpression of miR-424 before induction of ischemia significantly reduced cerebral infarct size and brain edema. This improvement of brain injury was coupled to decreases in activated caspase-3, cytokine production and microglial proliferation (Zhao et al., 2013).

### Efferocytosis and phagocytosis

Efferocytosis is largely mediated by microglia in the CNS, and it is necessary for inflammation resolution. Microglia sense changes in the CNS environment and rapidly responds to alterations in the CNS by phagocytosis and secretion of inflammatory factors. Insufficient efferocytosis has been shown to worsen the outcome of neuroinflammatory diseases such as ischemic stroke and demyelination (Cantoni et al., 2015; Cai et al., 2019) For instance, STAT6 was shown to be activated in microglia of humans and mice that suffered a stroke and enhances efferocytosis, as well as protect neurons from death. *In vitro* and *in vivo* studies demonstrate that *Stat6* ^−/−^ microglia display reduced efferocytosis of neurons, causing the accumulation of dying/dead cells. This was also associated with an increase in pro-inflammatory cytokines (e.g., *Il6* and *Tnfa*) and reduced anti-inflammatory mediators (such as *Il10* and *Arg1*), compared to wild type microglia. In turn, *Arg1* was found to be necessary for STAT6-induced efferocytosis post-ischemic stroke (Cai et al., 2019). Interestingly, microglia efferocytosis and its ability to suppress inflammation can be enhanced through the activation of AXL receptor tyrosine kinase signaling by growth arrest-specific 6 (Gas6) in a mouse model of brain aneurysm (Tang et al., 2022). It is, thus, possible that Gas6 stimulation or administration may help enhance efferocytosis in other neuroinflammatory conditions.

Microglia phagocytosis is impaired in Alzheimer’s disease (AD), and is needed to clear Aβ plaques. Impaired microglial phagocytosis was recently shown to be stimulated by elevated level of HK2 in AD mice and humans. Deletion or inhibition of HK2 lowers the level of Aβ by enhancing phagocytosis and clearance of Aβ by microglia, eventually improving AD pathology and cognitive function (Leng et al., 2022). Downregulation of HK2 is thought to facilitate phagocytosis by stimulating *Lpl* expression. Microglia LPL was further shown to promote phagocytosis of Aβ, by allowing the cells to produce ATP, thus providing sufficient fuel for this energy-demanding process (Leng et al., 2022). TREM2 is another regulator of *Lpl*, and has also been thought to regulate efferocytosis/phagocytosis. In a cuprizone-induced demyelination brain injury model, *Trem2* deficiency leads to defects in microglia morphology and proliferation, resulting in the inability of microglia to clear myelin debris. This ultimately results in accumulation of myelin debris in the CNS, more axonal damage, and subsequent behavioral defects (Cantoni et al., 2015).

## Discussion

Macrophages play various critical, sometimes divergent roles in most tissues. Many studies which involve the inhibition of macrophage recruitment and function are often limited to one tissue. A major challenge in targeting macrophages *in vivo* is the concern of affecting these critical functions in a non-diseased tissue. It is evident from this review that the discussed metabolic conditions have shared drivers and themes. For instance, the involvement of monocytes, and specifically CCR2-dependent recruitment, was shown to promote metabolic disease. While limiting macrophage recruitment (*via* inhibition of CCR2-CCL2 interaction, for instance) during disease initiation and progression can potentially result in reduction of disease severity, it may also impair disease resolution and other homeostatic functions. This is crucial, since by the time patients are diagnosed and treated, they already have established, complex, medically overt conditions, hence, disease resolution is the ultimate goal. Thus, it is imperative to better understand the unique signals activated during disease resolution and leverage them to find novel therapies. Additionally, many of these pathways are important to fight against infections, making them irrelevant as therapies. This is most evident in the case of IL-1β, a major promoter of metabolic inflammation, and an obvious therapeutical target for these conditions. Indeed, the CANTOS study showed that anti-IL-1β treatment reduces cardiovascular events. That said, it also increased lethal infections, and thus cannot be considered as a therapeutic (Soehnlein and Libby, 2021). Possibly, a more promising approach is upregulation of inflammation curbing cues, including anti-inflammatory cytokines and pro-resolving mediators. A combination of several inflammation resolving signals may also be considered, controlling the inflammation on one hand, and inducing efferocytosis and metabolic shifts on the other hand.

Since many metabolic diseases share etiology (i.e., obesity), and obese individuals typically suffer from >1 comorbid condition (Khaodhiar et al., 1999; Lim and Boster, 2022), it is critical to evaluate multiple tissues in any one study. Better understanding of the systemic and local effects of any treatment will greatly assist in evaluating how it will translate to therapy. Additionally, this might provide a treatment for several conditions concomitantly. In fact, such interventions already exist (albeit with very low adherence), with mechanisms of action largely unknown. These include weight loss and exercise. Understanding how these methods exert their benefits will be transformative in the treatment of obesity complications.

Another approach that might be beneficial is targeted therapy, affecting only relevant tissues and cell types locally. With that said, a major complexity of obesity is its detrimental effects on cell types and tissues that are distributed throughout the body. Some examples from this review are the nervous system, adipose and vasculature, which are widespread. It is possible that these tissues regulate and are in turn controlled by organ microenvironments distinctly. For instance, neurons innervating the liver, gut, kidney, etc. have similar or discrete influence across tissues in obesity, and how this affects local immunity and homeostasis is unknown. Through more investigation we are hopeful that some of these issues will be resolved in the upcoming years to help combat modern diseases.
